# Cobalt induced electronic and defect structure modulation in nickel iron layered double hydroxides for electrochemical, photo-electrochemical and photocatalytic water splitting

**DOI:** 10.1039/d6ra08160f

**Published:** 2026-10-27

**Authors:** Annamalai Kamalakkannan, Salma Machiche, Balakumar Subramanian, Sasirekha N. R, Mohamed Essalhi, Otman Abida

**Affiliations:** a African Sustainable Agriculture Research Institute (ASARI), College of Agriculture and Environmental Sciences (CAES), Mohammed VI Polytechnic University (UM6P) Laayoune Morocco otman.abida@um6p.ma annamalai.kamalakkannan-ext@um6p.ma; b National Centre for Nanoscience and Nanotechnology, University of Madras Chennai India; c Catalysis Laboratory, Centre of Advanced Study in Crystallography & Biophysics, University of Madras Chennai India

## Abstract

Multifunctional materials capable of meeting the growing demand for clean energy are being actively pursued as sustainable alternatives to fossil-fuel-based resources. Subtle modifications to the crystal structure can significantly alter the electronic configurations of such materials, enabling enhanced performance across multiple catalytic processes. In this context, we report the optimal incorporation of Co into the NiFe LDH nanostructure to achieve versatile catalytic functionality. Structural analysis reveals the formation of hierarchical architectures while offering redox-active hydroxyl-rich interfaces beneficial for electrocatalytic and photocatalytic reactions. Furthermore, the substitution of Co ions in the LDH layers promotes Co 3d–O 2p hybridized intermediate states, leading to electronic band structure modulation characterized by the optical band gap narrowing from 2.36 eV to 1.88 eV and formation of a plausible p–n heterojunction as evidenced from UV-vis diffuse reflectance spectroscopy and Mott–Schottky (MS) analysis, respectively. Owing to these synergistic structural and electronic effects, the optimized NiCoFe (1 : 2 : 1) configuration exhibits enhanced photoelectrocatalytic hydrogen evolution (HER) and oxygen evolution (OER) activities, delivering low overpotentials of 155 mV for the HER and 350 mV for the OER, with Tafel slope values of 270 and 26.4 mV dec^−1^, respectively. Overall, for electrocatalytic water splitting, the same catalyst achieves an overpotential of 234 mV for the HER and 50 mV for the OER, accompanied by favourable Tafel slopes of 114 and 285.6 mV dec^−1^, respectively. Photocatalytic hydrogen evolution measurements using NiCoFe (1 : 2 : 1) LDH further revealed an average H_2_ evolution rate of 925 µmol h^−1^ g^−1^ in comparison with bare (NiFe LDH), which only had a rate of about 752 µmol h^−1^ g^−1^. These results demonstrate that cobalt-induced electronic and structural modulation in NiFe LDHs provides a promising platform for efficient multimodal water-splitting applications.

## Abstract 2

Cobalt substitution tunes the electronic and defect structure of NiFe LDH, and NiCoFe (1 : 2 : 1) delivers the best electrochemical, photoelectrochemical and photocatalytic water-splitting performance.

## Graphical abstract

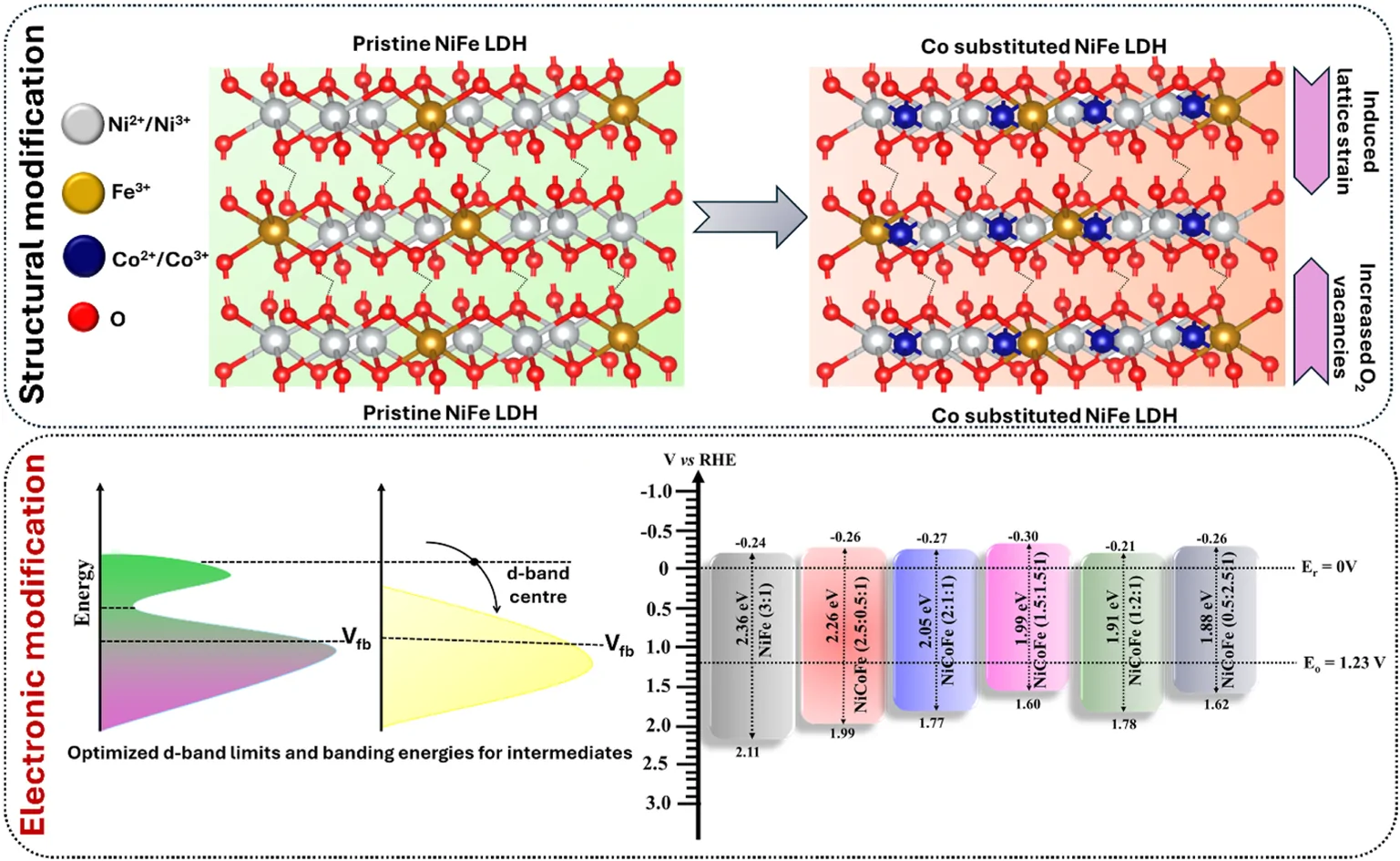

## 1 Introduction

To meet the growing demands for alternative green fuels, several technologies such as flow batteries, electrocatalytic fuel cells, photocatalytic and photo-electrochemical systems, and electrolyzers have been developed. The adoption of CO_2_ emission-free technologies is the primary focus for researchers and policymakers across different nations to achieve net zero by the year 2050.^1^ In this context, moving away from natural energy sources, hydrogen (H_2_) has been considered as an alternative fuel due to its environmental friendliness and higher energy density (142.35 MJ kg^−1^).^2,3^ In the H_2_ colour spectrum (production techniques), green hydrogen produced through electrolysis, backed by renewable energy sources like wind or solar energy tends to release ≤2 kg of CO_2_ per kg of hydrogen evolved.^4^ Coupled with high energy density and zero pollution free by-product formation, green H_2_ production using various technologies is being tapped for versatile commercial and industrial applications.^5^ Green H_2_*via* electrolysis,^6^ photo-electrocatalysis (PEC), photocatalysis,^7^ and proton exchange membranes (PEM),^8^ are some of the major technologies involved in the sustainable production of H_2_. However, each approach has its own bottlenecks, making it inefficient in term of cost effectiveness and stability. For instance, electrolysis, PEC water splitting and photocatalytic water splitting approaches have not reached the adequate solar to hydrogen (STH) percentage making them unsuitable for practical implications.^9^

Electrochemical and photo-electrochemical water splitting proceed through two half-cell reactions namely, the oxygen evolution reaction (OER) at the anode and the hydrogen evolution reaction (HER), at the cathode. OER is undergoing a four-electron transfer process, and sluggish reaction kinetics and high overpotential reduce the water-splitting efficiency.^10^ In heterogeneous photocatalytic water splitting, in addition to low STH efficiency, many semiconductors are unstable, suffer from rapid exciton recombination, and non-oxide materials like CdS, and ZnS, are often susceptible to photo corrosion, reducing their lifetime.^11^ Furthermore, commonly studied semiconductors such as TiO_2_,^12^ ZnO,^13^ and BiVO_4_,^14^ often have wide optical band gaps and inadequate band edge potentials, making them unsuitable as multifunctional catalytic materials for water splitting.

To circumvent these intrinsic limitations and maximize overall water-splitting efficiency, it is essential to explore the multifunctional properties of advanced nanomaterials. Developing a single, robust catalytic system, which can operate effectively under conventional electrochemical and light-driven conditions is highly desirable. Such multifunctional materials not only streamline the system design but also limits the practical implication costs. Hence, in the wake of this 3d transition metal-based layered double hydroxides (LDHs) have emerged as promising candidates. LDH materials often provide flexibility in tuneable chemical composition, bandgap, and band edge modifications, and remarkable stability.^15^ Amongst them, NiFe LDH is widely considered as a benchmark in the electrocatalysis for OER, due to its 2D layered structure, tunable composition, and exposed active surface sites. However, pristine NiFe LDH suffers from poor intrinsic electrical conductivity and restricted active sites, which severely affect the overall water splitting activity. Hence, to fine-tune the catalytic, optical, and electronic properties of this NiFe LDH, doping or substitution of a heteroatom into the crystal structure has have proven to be an effective strategy. As previously explained by Weibin Chen *et al.*, earth-abundant transition metals such as Co and Fe have been considered as alternatives to noble metals due to their unique 3d electronic structure.^16^ Furthermore, in alkaline media, too weak or too strong formation of the metal–OH bond could lead to large overpotential values, as the adsorption and desorption of oxygenated intermediates (˙O, ˙OH, ˙OOH) during OER catalytic activity is highly dependent on the bonding of surface metal active sites and intermediates.

Hence, a controlled combination of Ni, Co, and Fe are expected to provide an optimal bond strength between M–OH/O and M–H* for effective OER and HER activity respectively. Several bi- and tri-metallic LDHs have been developed by forming different compositions of Ni, Fe, and Co and OER have been achieve with reduced overpotential (*η*). For instance, Boyao Zhang *et al.* reported on the optimal Ni : Co : Fe ratio which helped in the synergistic improvement in efficient energy storage capability and electrocatalytic water splitting.^17^ Following this, Fe -incorporated NiCo LDH reported by Kyoung Ryeol Park *et al.* reported on the lower overpotential value of 215 mV @ 100 mV cm^−2^, modulating the electronic structure *via* shifting the d-band downwards, which in turn lowers the energy barrier of the rate-determining step.^18^ Hence, the interplay between these transition-metal compositions has been shown to modify the crystallinity, morphology, and electronic modifications, which are crucial for the design of multifunctional catalysts for water-splitting applications. Although many studies have reported on the improved OER properties, the multifaceted properties of this ternary LDH have not been reported elsewhere. The novelty of the work lies in the systematic correlation of local electronic structural modulation, driven by optimal cobalt substitution, with the consequent formation of p–n heterojunction that synergistically boosts all three water-splitting models.

In this study, the strategic synthesis and electronic-structure modulation of Co-substituted NiFe LDH nanostructures have been extensively carried out. By systematically varying the cobalt concentration within the host NiFe LDH matrix, a layered ternary hydroxide has been formed, aiming to unlock multifunctional properties that enable high catalytic for water splitting. Comprehensive structural and optoelectronic analysis revealed that an optimal Ni : Co : Fe ratio of 1 : 2 : 1 induces a localized lattice strain, and morphological reorganization, yielding highly active 2D nanostructures with superior electrochemically active surface area. The incorporation of Co effectively narrowed the optimal optical band gap from 2.36 eV to 1.88 eV and upshifted the valence band maximum, thereby promoting highly efficient light harvesting, and charge transfer. X-ray photoelectron spectroscopy (XPS) confirmed distinct electron transfer from Ni and Fe sites to Co sites, establishing strong electronic hybridization that, in turn, reduces the recombination by acting as active trapping centers. Consequently, the optimal ratio NiCoFe (1 : 2 : 1) electrocatalysts exhibited significantly lower overpotentials, *η*_10_ = 50 mV *vs.* RHE for OER and *η*_10_ = 234 mV *vs.* RHE for HER, whereas NiFe (3 : 1) composition provides a higher overpotentials of *η*_10_ = 440 mV *vs.* RHE for OER and *η*_10_ = 323 mV *vs.* RHE for HER. Under light-driven electrocatalysis, the photocurrent density reached a maximum of 7 mA cm^−2^, achieving a remarkable 8.4-fold enhancement in comparison with bare NiFe (3 : 1), alongside a robust photocatalytic evolution rate of 925 µmol h^−1^ g^−1^. These findings presented herein offer an insightful correlation on the structural and electronic interplay of ternary LDHs, providing a scalable blueprint for designing highly efficient, multifunctional green hydrogen catalysts.

## 2 Materials and methodology

### 2.1 Precursor materials

All the chemicals were procured from commercial sources and were used without further purification. Double-distilled water (DDW) was used during the synthesis and for washing purposes as well, alongside ethanol. Nickel(ii) chloride hexahydrate [NiCl_2_·6H_2_O] was procured from Alfa Aesar with a purity of 98%, iron(ii) chloride hexahydrate [FeCl_2_·6H_2_O] with a purity of 98% was procured from Thermo Scientific, and cobalt(ii) chloride hexahydrate [CoCl_2_·6H_2_O] with analytical grade purity was purchased from Alfa Aesar. Urea (ACS analysis grade) and Ammonium fluoride (NH_4_F – laboratory-grade purity) were used for pH control.

### 2.2 Characterization tools

X-ray diffraction analysis was carried out using Aeries bench top XRD (Malvern PANalytical), FTIR analysis was carried out using Nicolet iS50 (Thermo Fischer scientific). Morphological analysis *via* FE-SEM was carried out using Sigma HV (Carl-Zeiss). UV-visible Diffuse Reflectance Spectroscopic (UV-DRS) analysis was carried out using Lambda 850 spectrophotometer (Perki Elmer). Electrochemical analysis was carried out using a single channel potentiostat/galvanostat 350 M (Corrtest instrumentations). Photo-electrochemical analysis was carried out using an electrochemical workstation (CHI6097E, Sinsil International, USA), fitted with a 100 W Xenon lamp solar simulator (Photo Emission Tech Inc). Gas evolution analysis was carried out using Gas chromatography, GC-2014 (Shimadzu), fitted with TCD (Thermal conductivity detector). Steady state PL and lifetime analysis were carried out using Fluorobog-FL3-11 (Jobin Yvon). Electron Paramagnetic Resonance Spectroscopy (ESR) was carried out using JEOL Model JES FA200 instrument. Inductively coupled plasma mass spectroscopy (ICP-MS) was carried out using Thermo Scientific iCAP TQ ICP-MS. X-ray photoelectron spectroscopy was carried out using K-Alpha-KAN9954133 (Thermo Scientific).

### 2.3 Synthesis methodology

In this work, the hydrothermal synthesis environment was used to prepare NiFe LDH and Co-substituted NiFe LDH nanoparticles (NPs).

Typically, to prepare NiFe (3 : 1) composition, 0.2674 g (1.125 mmol) of NiCl_2_ and 0.1014 g (0.375 mmol) of FeCl_2_ were dissolved in 30 ml of ultrapure water and stirred until complete dissolution. To this solution, 0.3 g of urea, and 0.2222 g of NH_4_F were added for pH control and were left to stir for 4 hours. This solution was transferred to a 50 ml autoclave fitted with a Teflon-lined chamber and kept in the oven at 160 °C for 4 hours. After the reaction time and cooling down of the autoclave, the supernatant was carefully removed, and the precipitated particles were washed thoroughly with ethanol and ultrapure water alternatively, followed by their drying process at 60 °C in a hot air oven for overnight. For Co-substituted NiFe LDH, the above procedure was carried out, but with the inclusion of different molar ratios of CoCl_2_. The exact amounts of precursor salts used to prepare the samples are provided in Table S3, and Fig. 1, which represent a schematic illustration of the synthesis methodology.

**Fig. 1:**
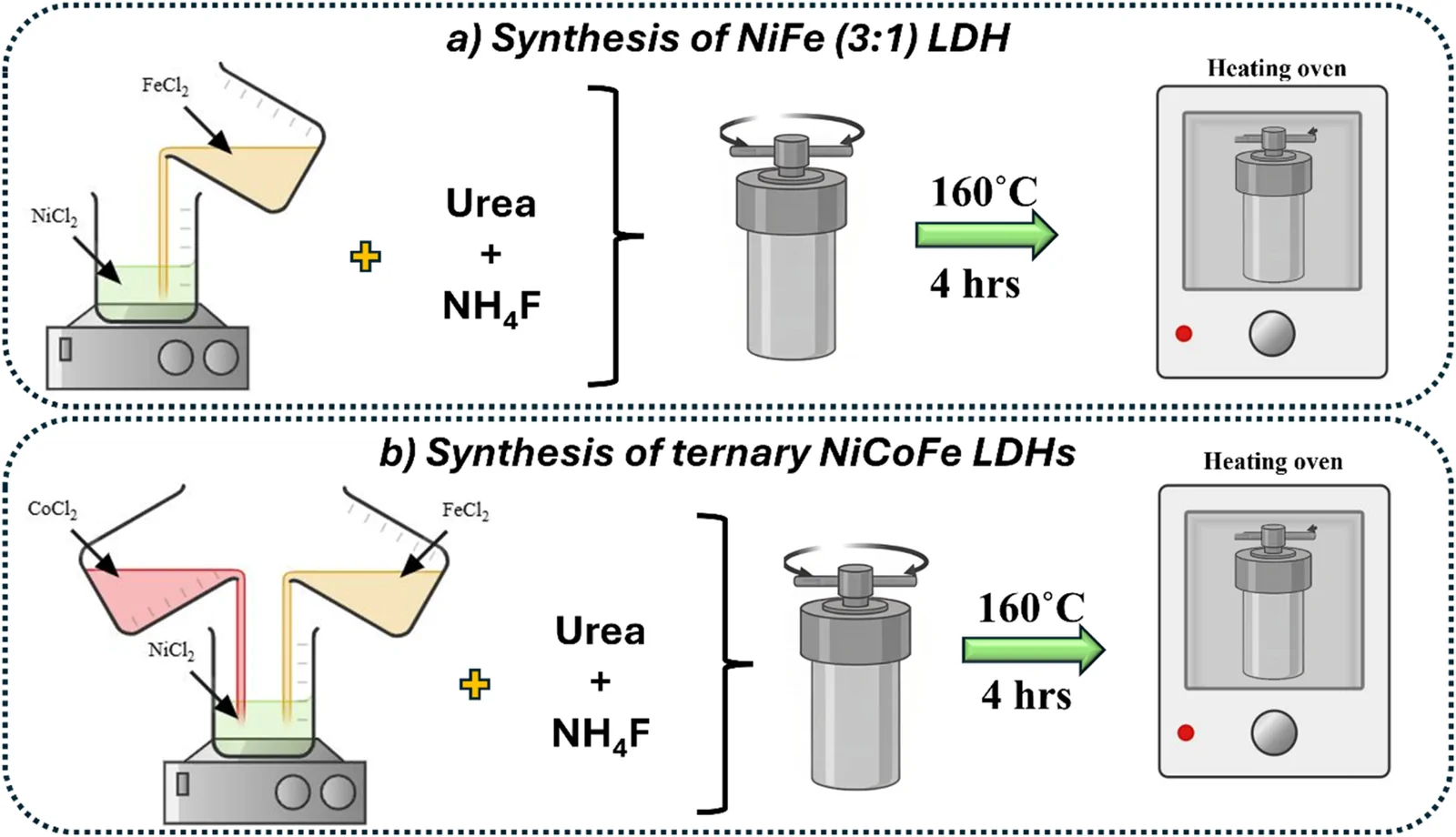
Schematic illustration of the synthesis of NiFe (3 : 1) LDH and Co-substituted NiFe LDH nanostructures.

### 2.4 Electrochemical analysis

The electrochemical water-splitting analysis was carried out in a three-electrode set-up, where the working electrode was chosen with the catalyst-coated nickel foam as the working electrode, an Ag/AgCl reference electrode filled with saturated KCl solution as the reference electrode, Pt wire as the counter electrode, and 1 M KOH as the electrolyte solution. Herein, to prepare the catalyst ink, 7 mg of the catalyst, 2 mg of PVDF, and 1 mg of activated carbon, were ultrasonically agitated together in 100 µL NMP solution until a homogeneous solution was formed. From this homogenous solution, 20 µL was drop-cast on a nickel foam with the working area of 1 cm × 1 cm and was left to dry overnight at 60 °C, and the catalyst loading was around to be 1.4–2 mg cm^−2^.

Prior to beginning the experiment, the electrolyte solution was purged with N_2_ to remove the dissolved oxygen. The Linear Sweep Voltammetry (LSV) analysis for oxygen evolution reaction (OER), was carried out on the potential window of 1.5 to 0 V *vs.* Ag/AgCl, and for hydrogen evolution reaction (HER), the potential window was fixed between 0 to −0.9 V *vs.* Ag/AgCl, with the scan rate of 10 mV sec^−1^. The Tafel slope was deduced from LSV, using the equation, *η* = *a* +*b* × log_10_*j*, where *b* is the Tafel slope (mV per decade), and *j* is the current density. The values are reported as such and *iR* compensation was not carried out. The electrochemical active surface area (ECSA) was estimated by running cyclic voltammetry (CV) with various scan rates of 20, 40, 60, 80 and 100 mV s^−1^ in the non-faradic region between 0 and 0.1 V for OER and −0.5 and −0.6 V for HER. The Nyquist plot was derived in the detection frequency range of 0.1 Hz to 10^6^ Hz with the amplitude of 20 mV. The detected potentials were converted to the reversible hydrogen scale (RHE) using the Nernst equation as given below,
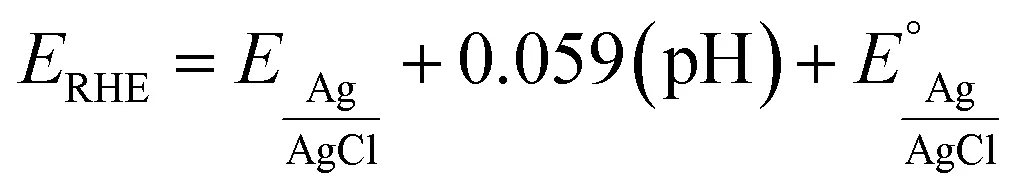
Herein, 
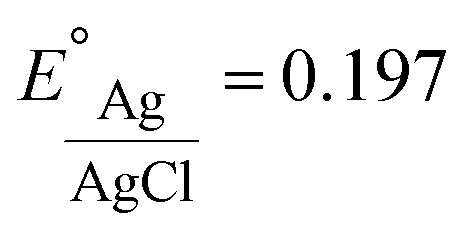
*vs.* NHE.

The ECSA was calculated using the below equation,
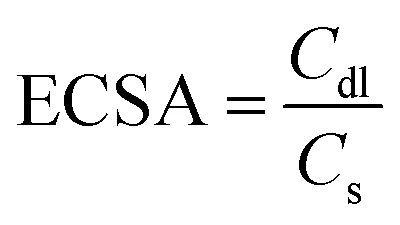
In the case of NiFe LDH (alkaline), the *C*_s_ value was considered to be 0.040 mF cm^−2^. Furthermore, the turnover frequency (TOF) was estimated from the ECSA, assuming that all surface sites are catalytically active. By standard approximation, the number of active sites were estimated by the below relation,Number of active sites (*N*) = ECSA × 1.6 × 10^15^Total current, *I* = *j* × *A*_geo_where *j* is the current density. The turnover frequency (TOF) was estimated by converting the number of reactant molecules per active site per second. For OER,
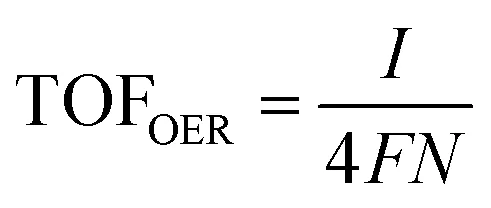
And for HER,
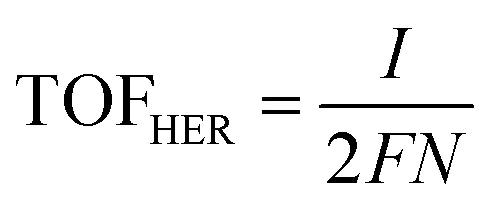
Here, *F* is the Faraday's constant = 96 485.3 C mol^−1^; 4 is the number of electrons that are required for the generation of one oxygen molecule; 2 is the number of electrons required for the production of one hydrogen molecule, and *N* is the number of active sites.

### 2.5 Photo-electrochemical analysis

The photo-electrochemical (PEC) analysis was carried out in a three electrode PEC cell, where the working electrode was chosen as the catalyst coated FTO plate, Pt wire as the counter electrode, Ag/AgCl as the reference electrode, and 0.5 M Na_2_SO_4_ was used as the electrolyte solution. Herein, the catalysts were drop-cast onto the FTO plate in a working area of 1 cm × 1 cm. Typically, to prepare the catalyst ink, 15 mg of the prepared catalyst was ultrasonically agitated for 30 min in the presence of 180 µL DDW, 35 µL Nafion (5%), and 200 µL isopropanol. The homogenous catalyst ink was subsequently drop-cast on the FTO plate and left to dry overnight in open air.

The experimental conditions were followed as described in the earlier reported work.^19^

### 2.6 Photocatalytic H_2_ evolution analysis

The evolution of H_2_ was carried out, where 10 mg of the prepared sample was added to the solution containing 40 ml DDW and 10 mL TEOA in a round-bottom flask. This solution was purged with N_2_ to remove dissolved oxygen and was sealed with a rubber septum to entrap the evolved gases. The sealed setup was degassed by a vacuum pump to remove trace gases before illumination. This setup was illuminated by a 500 W tungsten lamp for a reaction period of 3 hours, and after every one hour, the gas molecules were injected out using a 1 mL gas-tight syringe and injected into the TCD column of the GC instrument with N_2_ as the carrier gas.

## 3 Results and discussion

### 3.1 Phase evolution, bonding characteristics, and surface morphological analysis

The crystallographic phase confirmation of NiFe LDH (3 : 1) and NiCoFe LDH with varied Ni : Co : Fe ratios was examined using the X-ray diffraction spectroscopic technique, and the attained spectra are shown in Fig. 2(a).

**Fig. 2:**
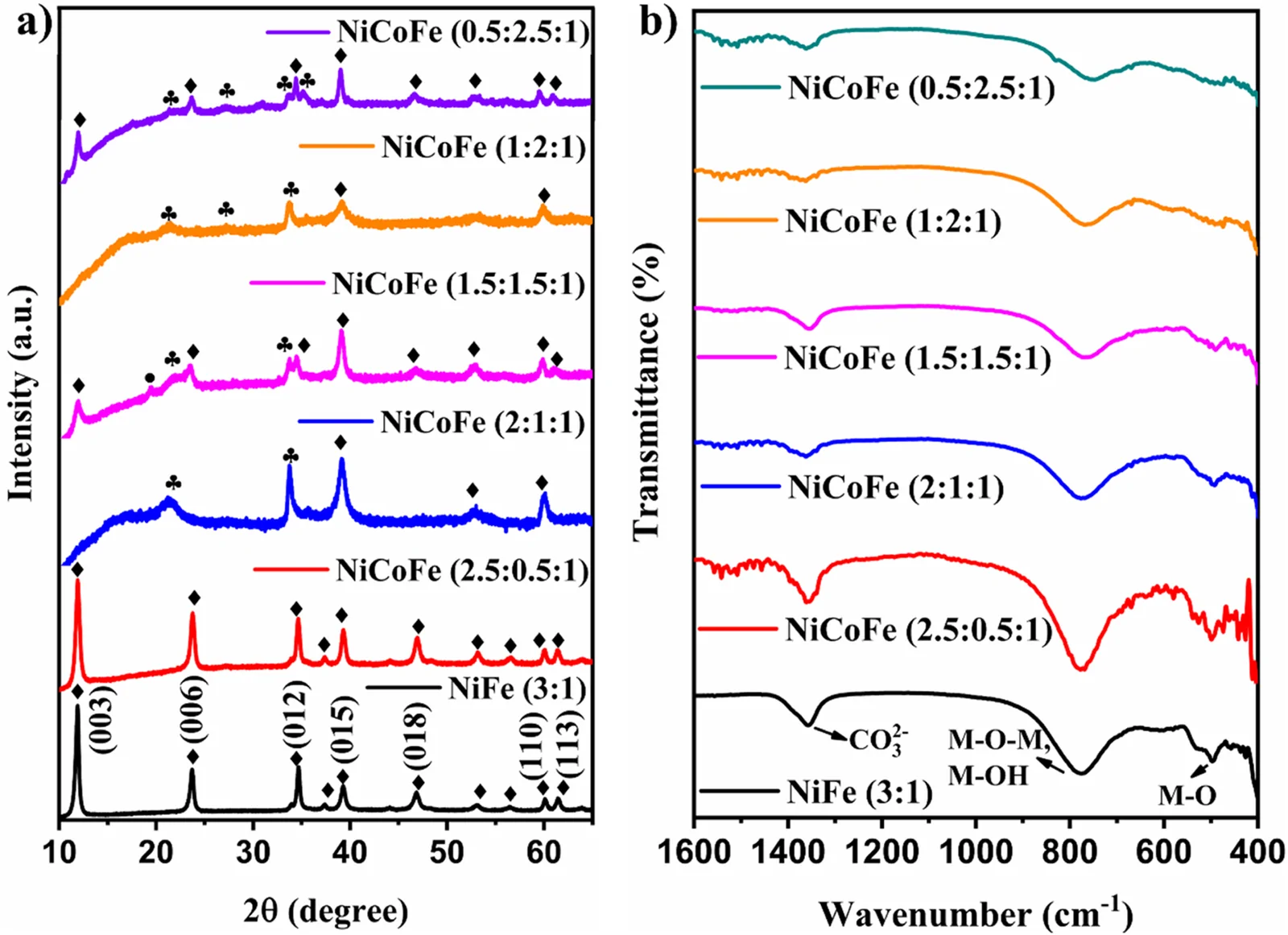
(a). X-ray diffraction profile of NiCoFe LDH; (b). Vibrational mode analysis of NiFe LDH and NiCoFe LDH from FTIR spectra.

The XRD spectrum of NiFe (3 : 1) composition with Ni/Fe ratio of 3 : 1, reveals the formation of diffraction peaks at, 11.46, 23.68, 34.67, 39.28, 46.84, 60.12, and 61.46 corresponding to the lattice planes of (003), (006), (012), (015), (018), (110), and (113). These peak positions confirm the formation of nickel iron carbonate hydroxide hydrate (ICDD # 00-049-0188) with a hexagonal crystal structure represented ♦ in the XRD spectrum. Generally, in LDH nanostructures, the sharp (003) and (006) planes correspond to the crystalline long order arrangement along the *c*-axis direction. With minimal inclusion of cobalt ions (Ni : Co : Fe = 2.5 : 0.5 : 1), the crystal structure was minimally disrupted and resembled that of the NiFe (3 : 1) LDH nanostructure, maintaining its long-ordered layered structure. However, for intermediate Co substitution, such as Ni : Co : Fe – 2 : 1 : 1, 1.5 : 1.5 : 1, and 1 : 2 : 1, there was a noticeable decrease and broadening, or, in some cases, disappearance of the (003) plane, which suggests lattice distortion and structural disorder within the brucite-like layers. As reported by Lorenzo Vigna *et al.*, this suggests a short layer stacking along the *c*-direction.^45^ When the cobalt inclusion is augmented (Ni : Co : Fe – 0.5 : 2.5 : 1), the emergence of cobalt iron carbonate hydroxide hydrate with a similar hexagonal structure was observed, as denoted by 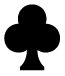 in the XRD spectrum, which confirms the incorporation of Co^2+^ ions into the NiFe frameworks. At this composition, Co is therefore no longer accommodated only within the NiFe hydroxide layers but partially segregates into a separate CoFe LDH phase, so that NiCoFe (0.5 : 2.5 : 1) is better described as a NiFe/CoFe LDH composite. For the lower and intermediate compositions (2.5 : 0.5 : 1 to 1 : 2 : 1), no separate Co-containing phase was resolved, and Co is considered to substitute for Ni in the octahedral sites of the brucite-like layers, while the interlayer galleries remain occupied by carbonate anions, as indicated by the FTIR band at 1357 cm^−1^ discussed below and by the basal spacing of NiFe (3 : 1) (*d*_003_ ≈ 0.77 nm). However, since NiFe and CoFe carbonate LDHs are isostructural with nearly coincident reflections, and the basal reflections of NiCoFe (1 : 2 : 1) are strongly broadened, nanoscale Co-rich domains in this sample cannot be excluded on the basis of XRD alone.

The apparent domain size (*D*), microstrain (*ε*) and dislocation density (*ρ*) were calculated using the equations given below,
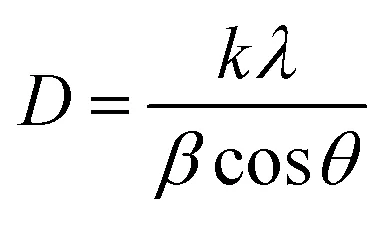

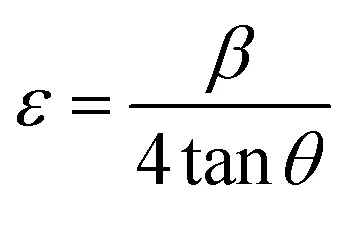
*ρ* = 1/*D*^2^where *D* is the apparent domain size, *k* is the shape factor (0.9), *λ* is the wavelength of X-ray used (Cu – *K*_λ_ = 1.54 Å), *β* is the full width half maximum (FWHM) of the reflection, *θ* refers to the diffraction angle, *ε* denotes the lattice microstrain, and *ρ* refers to the dislocation density. Herein, the *k* = 0.9, is interpreted as apparent domain size, where the (003) reflections corresponds mainly to the thickness of the layer stack along the *c*-axis. Since *k* value depends on the crystallite shape and *hkl* reflections, the decrease in *D* and loss of basal reflections with increasing Co content indicate smaller coherent domains and reducing stacking order. The attained metrics are tabulated in Table 1, the pristine NiFe (3 : 1) LDH exhibited an larger *D* value of up to 18.13 nm with higher microstrain value of 0.0072, and as the Co concentration increases in the ternary NiCoFe, the apparent crystallite domain size decreases, with an increase in microstrain and dislocation density with its highest reaching up to 0.0221 and 18.96 × 10^−5^ respectively for NiCoFe (1 : 2 : 1) sample. This suggests an extensive substitution of dissimilar metal ions ideally creating an internal stress and plausible defect sites or dislocations within the crystal structure, hindering the long-range crystalline ordering as observed from the XRD peak profile. Nevertheless, for an equal ratio of Ni/Co sample 1.5 : 1.5 : 1 sample, and a higher concentration of Co sample *i.e.*, NiCoFe (0.5 : 2.5 : 1), the apparent crystallite domain size increases again, whilst the strain and dislocation density decrease. This might be attributed to the stress relief provided by the nucleation and growth of secondary carbonate hydroxide hydrate phase as identified in XRD.

**Table 1:** Crystallographic metrics from X-ray diffraction spectrum

Composition	Scherrer's equation		W–H plot		Dislocation density (*ρ*)
	Average apparent crystallite domain size (*D*) – nm	Microstrain (*ε*)	Average apparent crystallite domain size (*D*) – nm	Microstrain (*ε*)	
NiFe (3 : 1)	18.13	0.0072	30.14	0.0032	3.04 × 10^−5^
NiCoFe (2.5 : 0.5 : 1)	17.490	0.0075	19.52	0.0012	3.26 × 10^−5^
NiCoFe (2 : 1 : 1)	9.84	0.0186	3.33	−0.0156	10.31 × 10^−5^
NiCoFe (1.5 : 1.5 : 1)	14.13	0.0099	14.75	0.0007	5.00 × 10^−5^
NiCoFe (1 : 2 : 1)	7.26	0.0221	2.45	−0.0224	18.96 × 10^−5^
NiCoFe (0.5 : 2.5 : 1)	18.46	0.0081	14.00	−0.0014	2.93 × 10^−5^

Furthermore, the apparent crystallite domain size, was carried out using the Williamson–Hall (W–H) analysis for all the compositions, using the uniform-deformation model as described below,*β* cos *θ* = *kλ*/*D* + 4*ε* sin *θ**β* cos *θ* plotted against 4sin *θ* is shown in Fig. S8; the slope gives the microstrain (*ε*), and the intercept gives the apparent crystallite domain size (*D*), and the attained values are tabulated in Table 1. Some compositions of Co-included NiFe LDHs display negative W–H slopes, which are not physically meaningful; however, this arises from anisotropic size broadening of the (003) reflection in the plate-like LDH crystallites. However, the decrease in lattice strain from 0.0032 for bare NiFe (3 : 1) LDH is attributed to stress relief by the secondary cobalt-based carbonate hydrate phase.

The chemical environment of the prepared nanostructures was carried out using FTIR under ATR mode. All the samples showed a similar spectrum to that of the binary composition of NiFe LDH (3 : 1), indicating all the compositions share an overall analogous structure of LDH, although there was Co substitution. The absorption peak at 1357 cm^−1^ corresponds to the antisymmetric stretching vibration of Co_3_^2−^ present in the interlayer space, bridging the bimetallic layers. The broader peak observed at 775 cm^−1^ is attributed to the vibrations of M–O–M and M–OH group (M = Ni/Co/Fe), and the peak at 500 cm^−1^ denotes the vibration of M–O group.

To visualize the compositional influence on the surface morphology of the prepared NiFe LDH and Co-substituted NiFe LDH, FE-SEM analysis was carried out, and the attained micrographs are shown in Fig. 3. The binary LDH with NiFe (3 : 1) composition displayed moderately aggregated, irregular flake-like sheets, and upon a slight Co substitution, a remarkable structural re-organization was observed. The samples with NiCoFe compositions of 2.5 : 0.5 : 1 and 2 : 1 : 1 exhibited 3D hierarchical, flower-like microspheres interconnected by 2D nanosheets. As the Co concentration was increased, the 3D architecture underwent a breakdown process and tended to form a smaller flake-like 2D nanostructures. This corelates with the smallest apparent crystallite domain size observed for NiCoFe (1 : 2 : 1) composition from the XRD metrics (Table 1), where the high lattice strain disrupts the growth of large adjoining sheets. However, for the highest Co concentration, with the composition NiCoFe (0.5 : 2.5 : 1), a heterogeneous morphology was observed, featuring rod-like structures alongside irregular sheets. This corresponds to the emergence of secondary cobalt iron carbonate hydroxide hydrate as detected in the XRD patterns. The EDX spectrum shown in Fig. S1 confirms only the presence of Ni, Co, Fe, carbon, and oxygen, indicating no impurity formation.

**Fig. 3:**
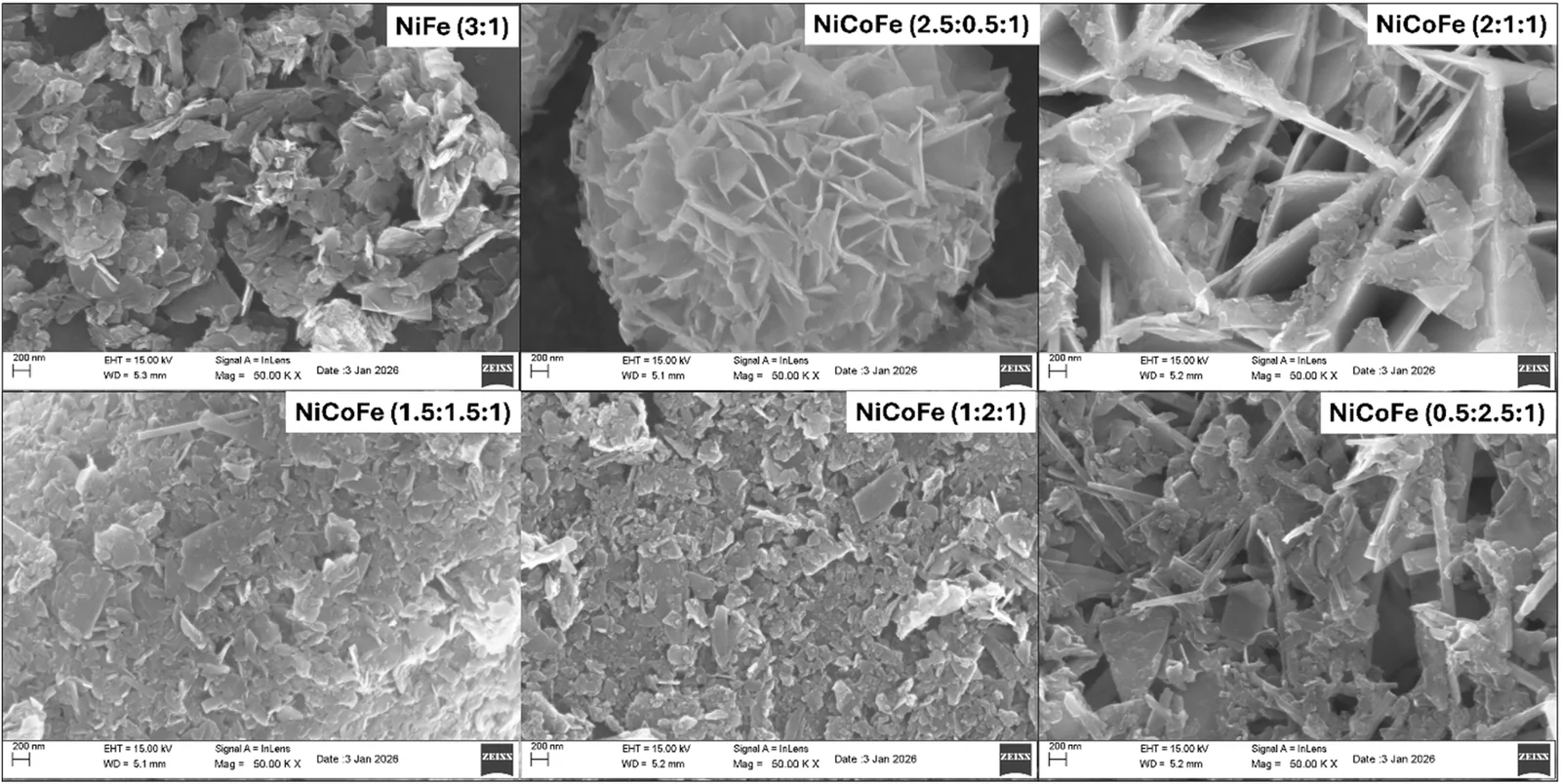
FE-SEM micrographs showing the microstructural evolution of NiFe LDH and cobalt substituted NiFe LDH architectures.

The corresponding atomic percentage table in the inset of each spectrum closely follows the intended precursor ratios, indicating compositional control during the synthesis. The gradual increase in Co concentration, corresponding to a decrease in Ni concentration, demonstrates the effective incorporation of cationic Co into the NiFe LDH matrix. Such tuning in the composition of NiFe (3 : 1) LDH, modulate the overall physio-chemical and electronic structure, which plays a crucial role in enhancing the catalytic activities of the prepared nanostructures. Since EDX is semi-quantitative and probes a limited sample volume, these values are taken as an indication of the overall composition rather than as proof of compositional homogeneity. Further support for the location of Co within the hydroxide layers at low and intermediate contents is provided by XPS (Section 3.8), where the Ni 2p binding energies shift to higher values with increasing Co content, indicating electron withdrawal from Ni through shared Ni–O–Co bonds, and by the shift of the EPR resonance (Section 3.9). Direct atomic-scale confirmation of the Co site by HRTEM/SAED or Co K-edge XANES/EXAFS was not available in this study, and the structural description is therefore based on the combined XRD, FTIR, EDX, XPS and EPR data.

To further substantiate the elemental composition and assess the stoichiometric variation associated with Co incorporation, ICP-MS analysis was performed on pristine NiFe (3 : 1), and optimized NiCoFe (1 : 2 : 1) LDH compositions. The ICP-MS results revealed Ni and Fe contents of 35.97% and 14.09 wt%, respectively for pristine NiFe (3 : 1), corresponding, to an experimentally determined Ni molar ratio of 2.43 : 1, slightly deviating from the nominal precursor ratio of 3 : 1. Furthermore, the Co included NiCoFe (1 : 2 : 1) composition exhibited Ni, Co, and Fe contents of 9.18, 32.59, and 15.42 wt% respectively, yielding an experimental Ni : Co : Fe ratio of approximately 0.57 : 2.00 : 1. The close agreement between experimental and nominal Co ratios confirms the substantial presence of cobalt in the synthesized material, while comparatively lower Ni fraction suggests composition-dependent differences in metal-ion incorporation and retention during hydrothermal synthesis. The observed variation in metal stoichiometry is also consistent with the Cobalt induced structural evolution identified through XRD spectrum, where NiCoFe (1 : 2 : 1) displays a dominant cobalt iron carbonate hydroxide hydrate phase. The quantitative ICP-MS results are summarized in Table S5, and this confirms the modification of the local electronic environment inferred from XPS and EPR analysis.

Dynamic light scattering (DLS) analysis was performed to determine the particle size and surface charge of the prepared NiCoFe LDH with varying compositional ratios. The particle size and surface charge values are shown in Fig. S3. The particle size estimation showed a significant decrease in the particle size from 922 nm for NiFe LDH to 503 nm and 604 nm for NiCoFe (2.5 : 0.5 : 1) and NiCoFe (1 : 2 : 1), respectively. The improved nucleation and reduced particle agglomeration led to the formation of a hierarchical nanostructure in NiCoFe (2.5 : 0.5 : 1). The fluctuation in particle size corresponds to changes in crystal growth kinetics and interparticle interactions. It should be noted that DLS measures the hydrodynamic diameter of particle aggregates in suspension rather than the size of individual nanosheets or crystallites; the variation between compositions therefore mainly reflects differences in aggregation, and the morphological conclusions of this work rest on the FE-SEM observations, with DLS used as a qualitative indication of dispersion. The moderately smaller particle size of NiCoFe (1 : 2 : 1) is highly beneficial for catalytic reactions, as smaller particles provide a higher surface area and more accessible active sites. Similarly, the corresponding surface charge distribution of the prepared LDH nanostructures are shown in Fig. S2(b). The highest zeta potential value for NiFe (3 : 1), NiCoFe (2.5 : 0.5 : 1), and NiCoFe (1 : 2 : 1) of about 39.46, 36.09, and 39.08, respectively, dictates a good dispersion stability and fewer agglomeration of particles. Amongst the prepared LDH nanostructures, the NiCoFe (1 : 2 : 1) tends to possess an optimal particle size and high zeta potential value, indicating the possession of high surface availability of active sites. These characteristics are favorable for enhancing catalytic and photo-electrocatalysts performance as stable and well-dispersed catalysts facilitate an efficient charge transfer and reactant accessibility.

### 3.2 Optical, band gap and electronic band offset analysis

The optical property analysis of the prepared NiFe and NiCoFe with varied Co compositions were carried out using UV-vis DRS. The resulting absorbance spectra are shown in Fig. 4(a).

**Fig. 4:**
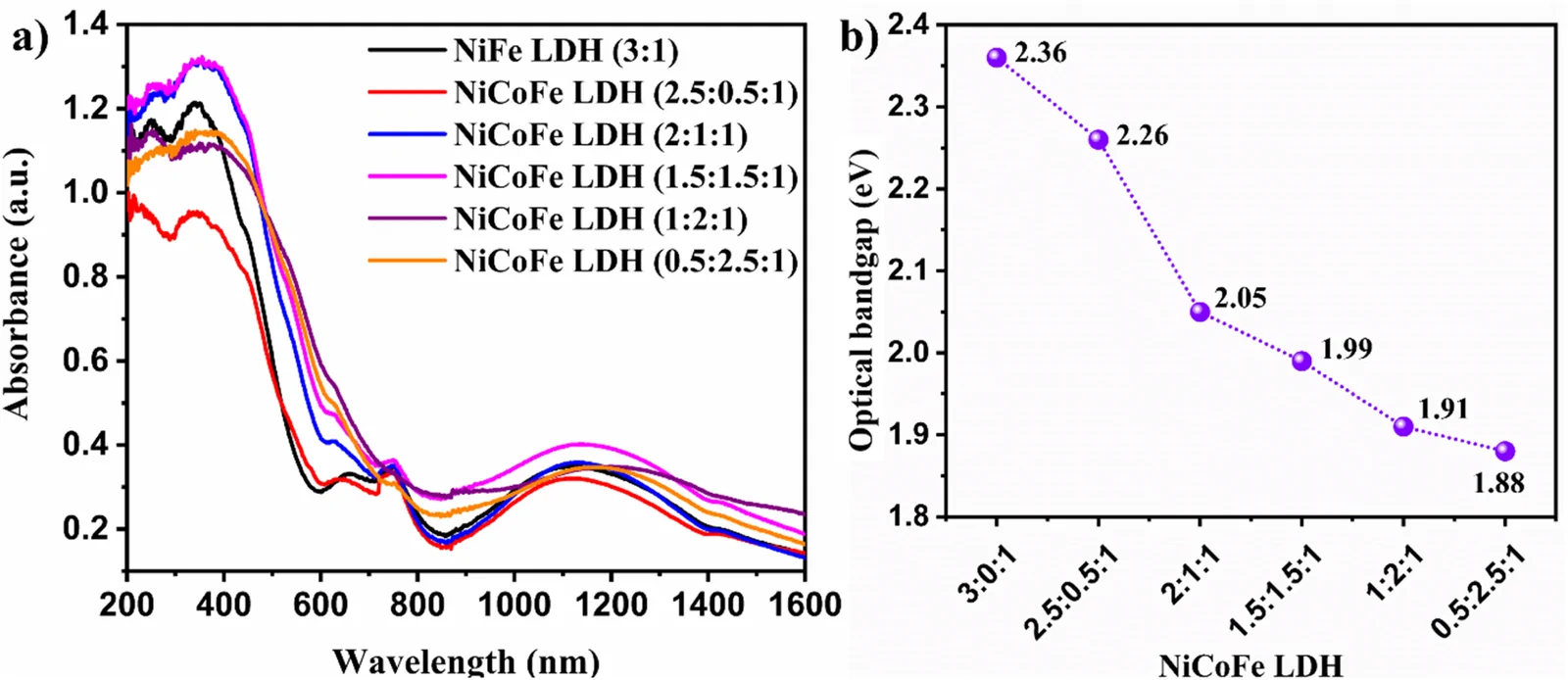
(a) UV-visible absorbance spectra of NiFe LDH and Cobalt substituted NiFe LDH; (b) estimated optimal band gap trend from Tauc plot.

Earlier reports by C. Suppaso *et al.*, on the absorbance spectra of NiFe LDH discussed that the peaks attained in the range of 200–400 nm correspond to the ligand to metal charge transfer (LMCT).^20^ The subsequent bands in the visible region and near-infrared regions were indexed to the d–d metal transition of octahedral Ni^2+^ (d^8^) from the NiFe LDH nanostructures. However, within the visible light range of 500–800 nm, the NiCoFe composition with a 1 : 2 : 1 ratio exhibited a slight red shift in the absorption spectrum, indicating its capacity to absorb more photons. This might be due to the stronger coupling of the Co into the Ni–O–Fe framework. The absorbance peak appearing at ∼780 nm is attributed to the spin-forbidden transitions ^3^A_2g_(*F*) → ^1^E_g_(*D*), which is in correspondence with the UV-DRS profile of NiAl LDH, and the minor peak near ∼650 nm attributes to the ^3^A_2g_(*F*) → ^1^T_1g_(*F*), spin forbidden d–d transitions (^1^E_g_, and ^1^T_1g_ are spin forbidden transition). This spin forbidden transitions to allow highly efficient optimal absorption of photons as well as providing a direct pathway for photo-generated carriers, maximizing initial efficiency. The fixed transition, and absence of metallic Ni^0^/Co^0^/Fe^0^ in XPS exclude a localized surface plasmon region, and the metal–metal charge transitions together with defect related substates contribute to the visible absorption and it's red shift with increasing Co content.^21,22^ Furthermore, these transitions minimize energy loss during the spin-flop process, paving the way for higher quantum efficiency in converting photon energy into chemical energy.^23,24^ The optical bandgap was calculated using Tauc plot using the equation as given below,*αhυ* = *A*(*hυ* − *E*_g_)^*n*^

Herein, *α* refers to the absorption coefficient, *A* is the arbitrary constant, *ν* refers to the frequency of light irradiated, and *E*_g_ refers to the optical band gap of the prepared material. The *n* value of the semiconductor is chosen as 2 or ½, depending on whether the transition is indirect or direct. For NiFe, *n* = ½ for direct transition and the Tauc plot represented between (*αhυ*)^2^*vs.* energy (*hυ*) is shown in the SI Fig. S3 and the extrapolated bandgap trend is shown in Fig. 4(b). Herein, *n* = ½ was chosen since the absorption edge of NiFe LDH is dominated by, O 2p → M (Ni^2+^/Fe^3+^) 3d ligand to metal charge transfer, which is electric dipole allowed. For such transitions, the absorption coefficient is proportional to the squared of dipole matrix element between the initial and final states multiplied by the joint density of states, which gives *α* ∝ (*hυ* − *E*_g_)^1/2^, when the matrix element is non-zero at the band edge. Hence, an indirect edge would require additional phonon assistance and would give a much weaker edge which is contradictory to momentum-conserving transitions.^25,26^ The pristine LDH (NiFe-3 : 1) displayed a bandgap value of 2.36 eV, and as the Co ions are incorporated into the NiFe, the optical band gap value appears to narrow down and reach a value of 1.88 eV for the highest Co content. These similar results were reported by J. Li *et al.,* wherein during the formation of NiCrFe LDH, the incorporation of Fe into the NiCr framework leads to metal-to-metal charge transfer resulting in a red shift in the absorbance.^27^ Similarly, the addition of Co ions to NiFe LDH and the hybridization of Ni 3d, Co 3d, and Fe 3d orbitals result in intermediate electronic states that enhance d–d transitions and metal–oxygen charge transfer transitions. From the absorbance spectrum (Fig. 4(a)), it can be clearly observed that NiCoFe (1.5 : 1.5 : 1) composition shows a sharp absorbance in the UV region and NiCoFe (1 : 2 : 1) composition tends to exhibit a broader absorbance in the UV and visible region. These characteristics suggests a strong improvement in the light harvesting capability facilitating a better photocatalytic activity.

To further analyze the electronic state modification in NiFe LDH upon Co inclusion, valence band (VB) XPS and Mott–Schottky (MS) plot were carried out, and the attained spectra are shown in Fig. 5(a) and (b), respectively. The VB potentials estimated from VB XPS show a shift in the valence band maximum (VBM) value towards the Fermi level for NiCoFe (1 : 2 : 1) composition (0.78 eV), whereas the binary composition NiFe LDH showed a VBM value of 1.21 eV. This upshift in the valence band confirms the electronic-level interaction among the Ni, CO, and Fe centers. Y. Dou *et al.*, reported on this upshift in the VBM during Al substitution in CoNi LDH due to the generation of oxygen vacancies, which reduces the coordination number of NiO_*x*_ and CoO_*x*_, and electronic density of Co/Ni sites due to the difference in the electron contribution from the outermost orbital.^28^ Subsequently, this upshift also corresponds to the strengthening of M–O interactions upon Co inclusion. The higher VB position, closer to the Fermi level, usually leads to faster hole-transfer kinetics, thereby improving OER characteristics and delaying recombination under light irradiation.

**Fig. 5:**
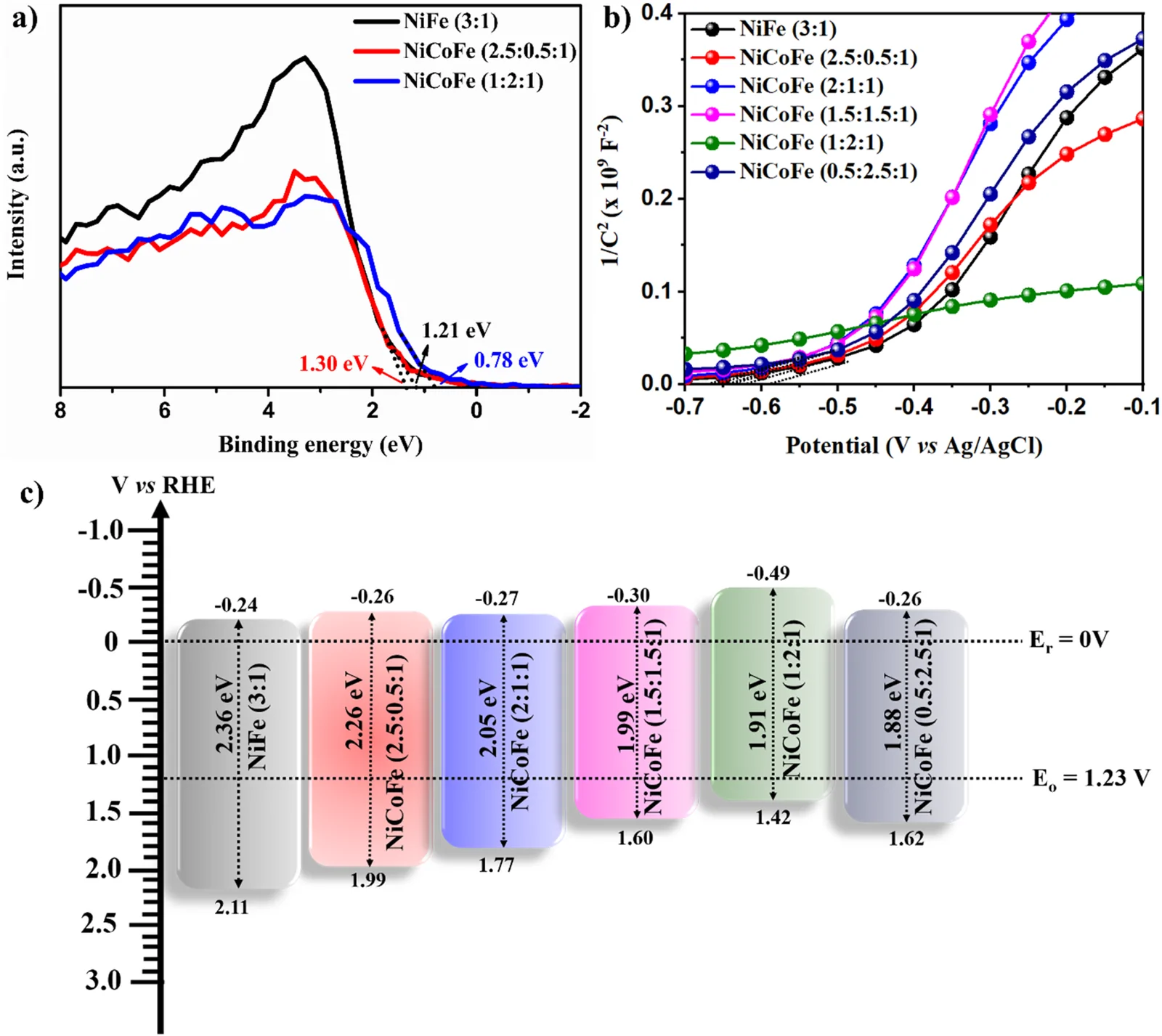
(a) Valance band spectra (VBS) from X-ray photo-electron spectroscopy; (b) Fermi level position (*V*_fb_) estimation from Mott–Schottky plot (MS) for NiFe and NiCoFe LDH samples; (c) schematic illustration of band edge positions extrapolated from MS plot.

The band structure of the prepared catalysts was constructed by determining the Fermi level (*V*_fb_) position from the MS plot, as shown in Fig. 5(c). To evaluate the density of carriers and *V*_fb_ position, slope, and *x*-intercept of the MS curve were carried out, respectively. Ideally, the positive slope values from the MS plot indicate the n-type characteristics of the prepared LDH nanostructures, and the negative slope indicates the p-type characteristics. Herein, from Fig. 5(c), it can be observed that a positive slope trend has been observed for binary NiFe (3 : 1) LDH indicating its n-type characteristics with electrons as the majority carriers. However, as the Co concentration increases, the profile of the MS curve shifts to an inverted V-shape (Fig. S4(a)). The NiCoFe (1 : 2 : 1) concentration indicates a sharp inverted V-shape curve indicating the formation of p–n heterojunction. These p–n heterojunction characteristics can be attributed to the p-type behaviour of CoFe LDH nanostructures, and from the XRD spectrum, it can be observed that for higher Co concentration, the existence of two phases (nickel iron carbonate hydroxide hydrate and cobalt iron carbonate hydroxide hydrate) was clear. Such p-type CoFe LDH-like domains are resolved as a separate phase by XRD at the highest Co content (0.5 : 2.5 : 1) and are likely present at the nanoscale in NiCoFe (1 : 2 : 1); the heterojunction assignment for this composition is therefore tentative. From the MS plot, the smaller slope values indicate higher carrier concentrations; NiCoFe (2.5 : 0.5 : 1) and NiCoFe (1 : 2 : 1) show minimal slopes, indicating higher carrier densities. The carrier concentrations (*N*_D_) were calculated using the equation given below,
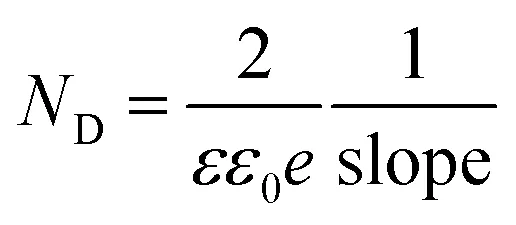


Herein, *ε*, *ε*_0_, and *e* indicate the relative permittivity, permittivity in vacuum, and electronic charge (1.6 × 10^−19^ C), respectively. The attained slope values and the calculated carrier density values are tabulated in Table 2. The NiFe LDH (3 : 1) sample shows a higher slope value, 0.826 and the equivalent *N*_D_ value was 1.71 × 10^21^ cm^−3^, and when on Co inclusion, the slope value is modified indicating the Co inclusion has modulated the charge carrier concentrations. The highest *N*_D_ values were found to be for NiCoFe (2.5 : 0.5 : 1) and NiCoFe (1 : 2 : 1), which are 2.25 × 10^21^ cm^−3^ and 8.98 × 10^21^ cm^−3^, respectively. This confirms that optimal Co substitution has effectively increased electron availability and facilitated faster charge transport.

**Table 2:** Band offset positions and carrier density metrics

Sample	*V*_fb_ (Ag/AgCl)	*E*_fb_ (RHE) = *V*_fb_ + 0.579	ECB = *E*_fb_ − 0.2	EVB = ECB + *E*_g_	Slope	Carrier density (ND) (cm^−3^)
NiFe (3 : 1)	−0.62	−0.041	−0.241	2.119	0.826	1.71 × 10^21^
NiCoFe (2.5 : 0.5 : 1)	−0.64	−0.061	−0.261	1.999	0.626	2.25 × 10^21^
NiCoFe (2 : 1 : 1)	−0.65	−0.071	−0.271	1.779	1.014	1.39 × 10^21^
NiCoFe (1.5 : 1.5 : 1)	−0.68	−0.101	−0.301	1.609	1.103	1.28 × 10^21^
NiCoFe (1 : 2 : 1)	−0.87	−0.291	−0.491	1.419	0.157	8.98 × 10^21^
NiCoFe (0.5 : 2.5 : 1)	−0.64	−0.061	−0.261	1.619	0.838	1.68 × 10^21^

The MS plots of pristine NiFe (3 : 1), and Co substituted NiCoFe (1 : 2 : 1) LDH recorded at different frequencies (1000, 2000, and 3000 Hz) are shown in Fig. S9(a and b). Both electrodes exhibit a near constant flat band potential (*V*_fb_) across the exhibited frequencies of −0.62 V and −0.87 V *vs.* Ag/AgCl for NiFe (3 : 1), and NiCoFe (1 : 2 : 1) respectively. The negligible frequency dependence of the extracted flat-band potentials suggest that the characteristic potential associated with the interfacial capacitance is stable within the investigated frequency range. With this, incorporation of Co induces a cathodic shift of 250 mV in the flatband potential compared to pristine NiFe LDH. This negative shift suggest substantial modification of the interfacial electronic structure and potential distribution following Co substitution. The more negative flat-band potential of NiCoFe (1 : 2 : 1) may suggest a more advantageous energy position for electron-driven reduction processes, assuming an n-type semiconductor response and a small distance between the flat-band potential and conduction-band edge. By strengthening the thermodynamic driving force for interfacial electron transfer, such electronic manipulation may lead to improved photoelectrochemical hydrogen evolution.

Furthermore, the *V*_fb_ values from the *x* intercept is shown in Table 2, where a negative shift in the *V*_fb_ position was observed for NiCoFe (1 : 2 : 1) in comparison with NiFe (3 : 1). Ideally, when the carrier density is high, a 0.2 V difference is considered during the estimation of conduction band minimum (CBM). Considering these, the estimated CBM and VBM values are tabulated in Table 2 and the visualized band structure is shown in Fig. 5(c). The negative conduction band potential and an optimal valence band potential of the prepared LDH nanostructures indicate their potential use in the bifunctional catalytic applications.

### 3.3 Electrochemical catalytic performance and stability

The electrocatalytic OER and HER kinetics were evaluated using 1 M KOH solution and the attained results are shown in Fig. 6(a)–(f). Fig. 6(a) shows the LSV curve of OER where NiCoFe (1 : 2 : 1) LDH exhibits a lowest overpotential value of 50 mV at 10 mA cm^−2^, which is significantly better than NiFe (3 : 1) with an overpotential value of 80 mV. The OER kinetics evaluated by Tafel slope is shown in Fig. 6(b), where the Tafel slope of NiCoFe (1 : 2 : 1) was attained to be 285 mV dec^−1^, whereas the binary NiFe (3 : 1) had a slope value of 340 mV dec^−1^. The higher Tafel slope value indicates the initial adsorption of hydroxide ions (M + OH^−^ → M − OH + e^−^) as the rate-determining step. The larger slope value also indicates limited charge transfer and a slow formation of the M–OH intermediate on the catalyst surface. The LSV curve for HER analysis is shown in Fig. 6(c) and their corresponding Tafel slope is depicted in Fig. 6(d).

**Fig. 6:**
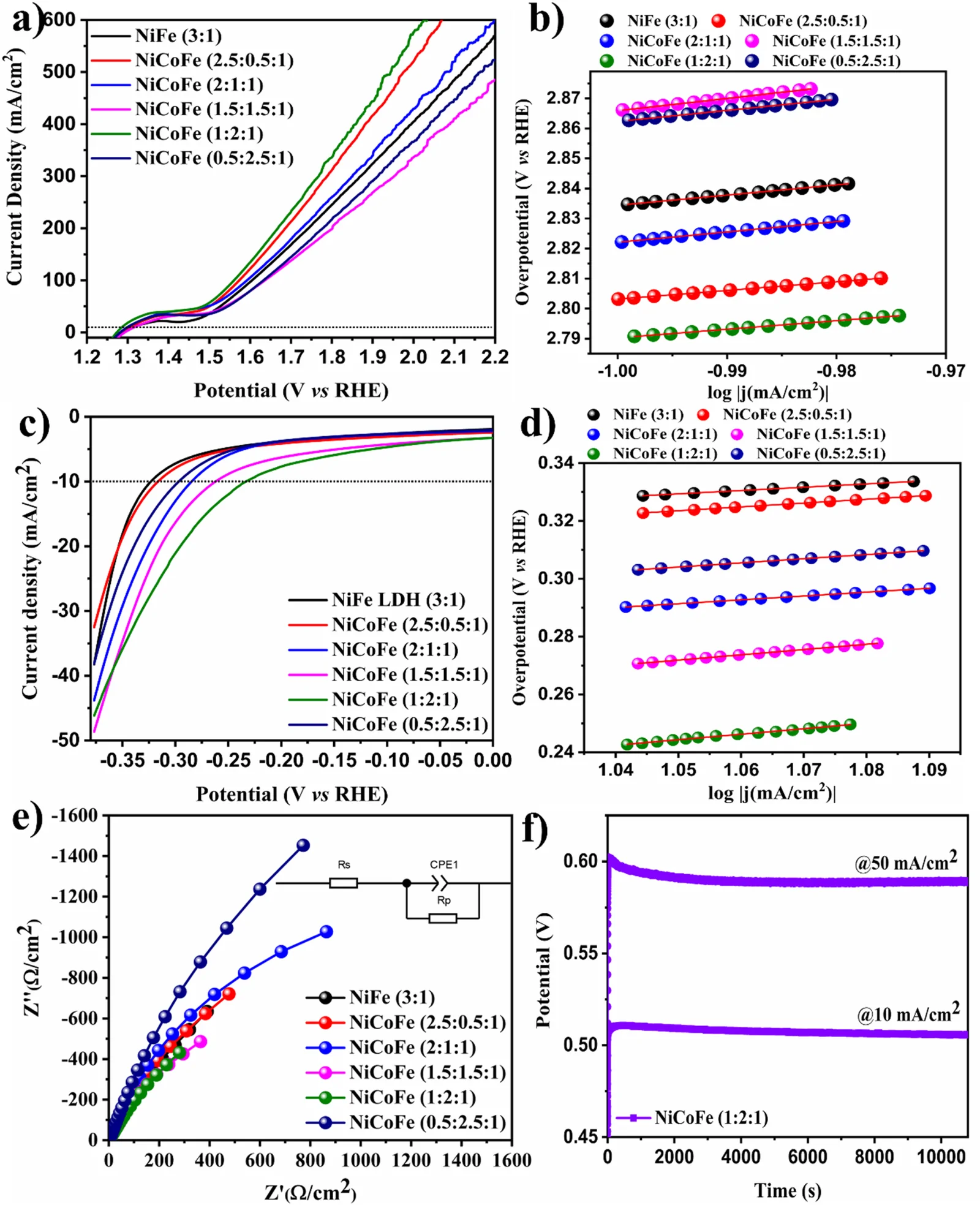
(a) Linear sweep voltammetry (LSV) curve for OER using NiFe and NiCoFe LDH @ 10 mV; (b) OER Tafel slope; (c) Nyquist plot using NiFe and NiCoFe LDH (inset) equivalent circuit; (d) linear sweep voltammetry (LSV) curve for HER using NiFe and NiCoFe LDH @ 10 mV; (e) HER Tafel slope; (f) chrono-potentiometric stability analysis @ 10 mA cm^−2^ and 50 mA cm^−2^.

The prepared catalyst with the composition NiCoFe (1 : 2 : 1) showed the lowest overpotential of 234 mV @ −10 mA cm^−2^ at a scan rate of 10 mV s^−1^, with a corresponding Tafel slope of 114 mV per decade. Herein, for HER, the rate determining step is the Volmer step where the reaction step goes by, M + H_2_O + e^−^ → M − H_ads_ + OH^−^. Followed by this, NiCoFe (1.5 : 1.5 : 1) showed the second-best minimal overpotential of 262 mV with the corresponding Tafel slope value of 182 mV dec^−1^. In comparison with NiFe LDH (3 : 1), with the overpotential and Tafel slope value of 323 mV and 190 mV dec^−1^, the Co substitution has highly influenced the charge transfer kinetics in the overall water splitting reaction. This could be attributed to the stronger p–n heterojunction formed at the catalyst surface with the composition NiCoFe (1 : 2 : 1). The complete metrics obtained during electrochemical water splitting are tabulated in Table 3.

**Table 3:** Electrochemical metrics from overall water splitting

Sample	OER		HER	
	Overpotential @ 10 mA–10 mV sec	Tafel slope (mV dec^−1^)	Overpotential @ −10 mA–10 mV sec	Tafel slope (mV dec^−1^)
NiFe (3 : 1)	80	340	323	190
NiCoFe (2.5 : 0.5 : 1)	70	288	316	132
NiCoFe (2 : 1 : 1)	60	344	284	133
NiCoFe (1.5 : 1.5 : 1)	90	410	262	182
NiCoFe (1 : 2 : 1)	50	285	234	114
NiCoFe (0.5 : 2.5 : 1)	70	368	297	143

Furthermore, to understand the physical and chemical barriers across the prepared catalyst, Nyquist plot was derived using electrochemical impedance spectroscopy (EIS) and the attained plot is shown in Fig. 6(d) with the equivalent circuit as the inset picture. The smallest semicircle formation ideally provides the lowest solution resistance (*R*_s_) and charge-transfer resistance (*R*_p_). From the initial analysis, it can be observed that, amongst the prepared samples, the catalyst with the composition 1 : 2 : 1, has provided a reduced *R*_s_ and *R*_p_ value. To further pinpoint the resistance values, the Nyquist plot was fitted to its equivalent circuit, and the attained values are tabulated in Table S41. The NiFe LDH showed the *R*_s_ value of 1.35 Ω, whereas NiCoFe (1 : 2 : 1) showed a decreased resistance of 1.2 Ω. Reports suggest that the introduction of Co^3+^ into NiFe LDH results in Co^3+^ acting as a Lewis acid and facilitating the formation of Ni^4+^. Furthermore, the existence of mixed valence states of Co^3+^/Co^2+^ creates a solid–solid interaction, accelerating the electron exchange during the OER and HER process by the NiCoFe (1 : 2 : 1) catalyst.^29^ The chronopotentiometry curves to determine the stability are shown in Fig. 6(f), indicating the stability of the prepared NiCoFe (1 : 2 : 1) at 10 mA cm^−2^ and 50 mA cm^−2^ as no considerable potential drop was observed after 3 h of analysis period. Furthermore, the FE-SEM micrographs (Fig. S6) of before and after stability (@ 50 mA cm^−2^) analysed substrates (Nickel foam) coated with NiCoFe (1 : 2 : 1) catalyst showed no change in the morphology confirming the stable structure of the prepared catalyst. Furthermore, the chronoamperometry analysis of NiCoFe (1 : 2 : 1) was carried at 0.5 V *vs.* Ag/AgCl for a long-term of about 32 hours, and the attained *i*–*t* curve is shown in Fig. S8a.The electrode exhibited a sustained anodic current response throughout the prolonged measurement, indicating its ability to maintain electrochemical activity under continuous polarization. The current density stabilized at 10 mA cm^−2^, and the absence of sustained decrease in current density over 32 h suggest a favourable operational stability of the NiCoFe (1 : 2 : 1) electrode.

The structural characteristics of the electrode after prolonged electrochemical operation was assessed using X-ray diffraction analysis, where before and after stability tests were carried out and is shown in Fig. S8b. Apart from the prominent nickel foam peaks at 43.9°, and 51.2°, the diffraction profiles show no obvious additional peaks after long term electrochemical testing. Overall, the sustained chronoamperometric response and post-stability diffraction characteristics demonstrate the favourable electrode level stability if NiCoFe (1 : 2 : 1) during prolonged anodic operation.

To further elucidate on the catalytic activity during HER and OER, the electrochemically active surface area (ECSA) was estimated using the linear relationship between the scan rates and Δ*j*, and is represented in the Fig. 7(a) and (b).

**Fig. 7:**
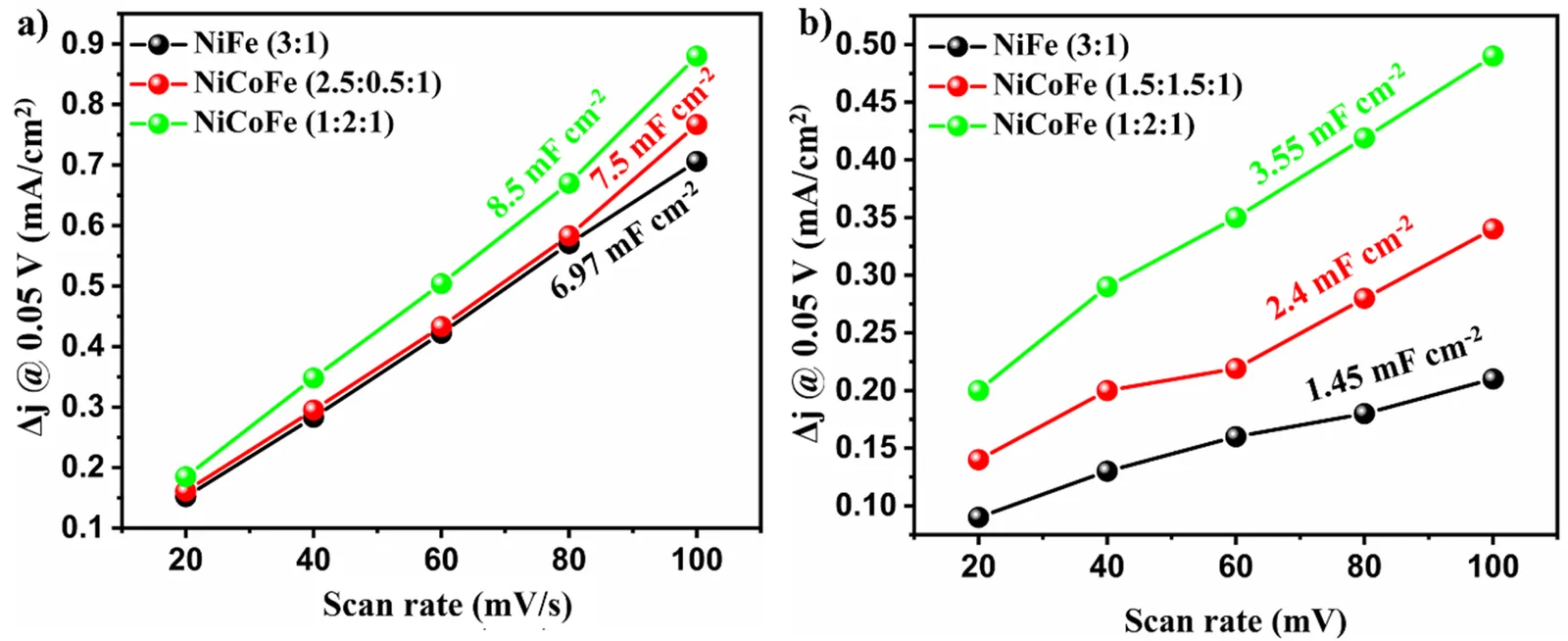
(a) Electrochemical active surface area for OER; (b) electrochemical active surface area for HER.

For OER and HER activity, the NiCoFe (1 : 2 : 1) exhibits a high *C*_dl_ value of 8.5 mF cm^−2^ and 3.55 mF cm^−2^, exhibiting the largest electrochemically active site. For OER activity, the attained value is greater than the previous reported composition of NiCo_1_Fe_1_ : NF by Wang *et al.*^30^ This proves that optimal Co incorporation has improved the density of active metal sites (Ni/Co/Fe). The enhanced ECSA is also attributed to the hierarchical, exposed nanosheet morphology, as depicted in FE-SEM micrographs, which facilitates improved electrolyte diffusion and charge transfer, as confirmed by EIS analysis. The TOF values were estimated based on ECSA assuming all electrochemically accessible sites are active. At a current density of 10 mA cm^−2^, the observed OER TOFs were determined to be 0.0560, 0.0531, and 0.0459 s^−1^ for NiFe (3 : 1), NiCoFe (2.5 : 0.5 : 1), and NiCoFe (1 : 2 : 1), respectively. The associated apparent HER TOFs were 0.5381, 0.3251, and 0.2198 s^−1^.

Despite NiCoFe (1 : 2 : 1) displaying relatively diminished apparent TOFs at the same current density, it showcases enhanced overall catalytic efficacy, as indicated by its reduced OER and HER overpotentials. This differentiation suggests that the improvement in geometric current responsiveness correlates with a larger estimated electrochemically accessible surface area and advantageous reaction kinetics, rather than a proven rise in the turnover rate per presumed active site. Moreover, as the active-site population was calculated based on a constant surface-site density and specific capacitance, the TOF values ought to be regarded as estimations reliant on the model. A conclusive assessment of intrinsic catalytic performance necessitates independently measured active sites and TOF analysis at a uniform applied overpotential.

### 3.4 Plausible electrocatalytic mechanism

Under typical electrochemical conditions, the Co substitution profoundly altered the overall charge distribution across the layered NiFe LDH matrix. The distinct electron transfer from Ni and Fe sites to Co sites, as confirmed from the high-resolution XPS spectra, effectively modulates the local electronic environment and shifts the d-band center of the transition metal active sites. Furthermore, the existence of mixed valence states, specifically the Co^3+^/Co^2+^ redox couple creates a strong solid–solid interaction that significantly accelerates electron exchange during the catalytic cycles.

During OER, Co^3+^ acts as a Lewis acid, facilitating the generation of highly catalytically active Ni^4+^ species. This electronic coupling optimizes the binding energies of the metal–oxygen intermediates, thereby boosting reaction kinetics by shifting the rate-determining step away from sluggish hydroxide adsorption towards much faster O–O bond formation or direct O_2_ desorption. Subsequently, in the HER pathway, electronic modulation of surface sites favors the Volmer step, thereby lowering the activation energy for water dissociation. The plausible mechanism involved in the electrocatalytic dissociation pathway of overall water dissociation is shown in Fig. 8.

**Fig. 8:**
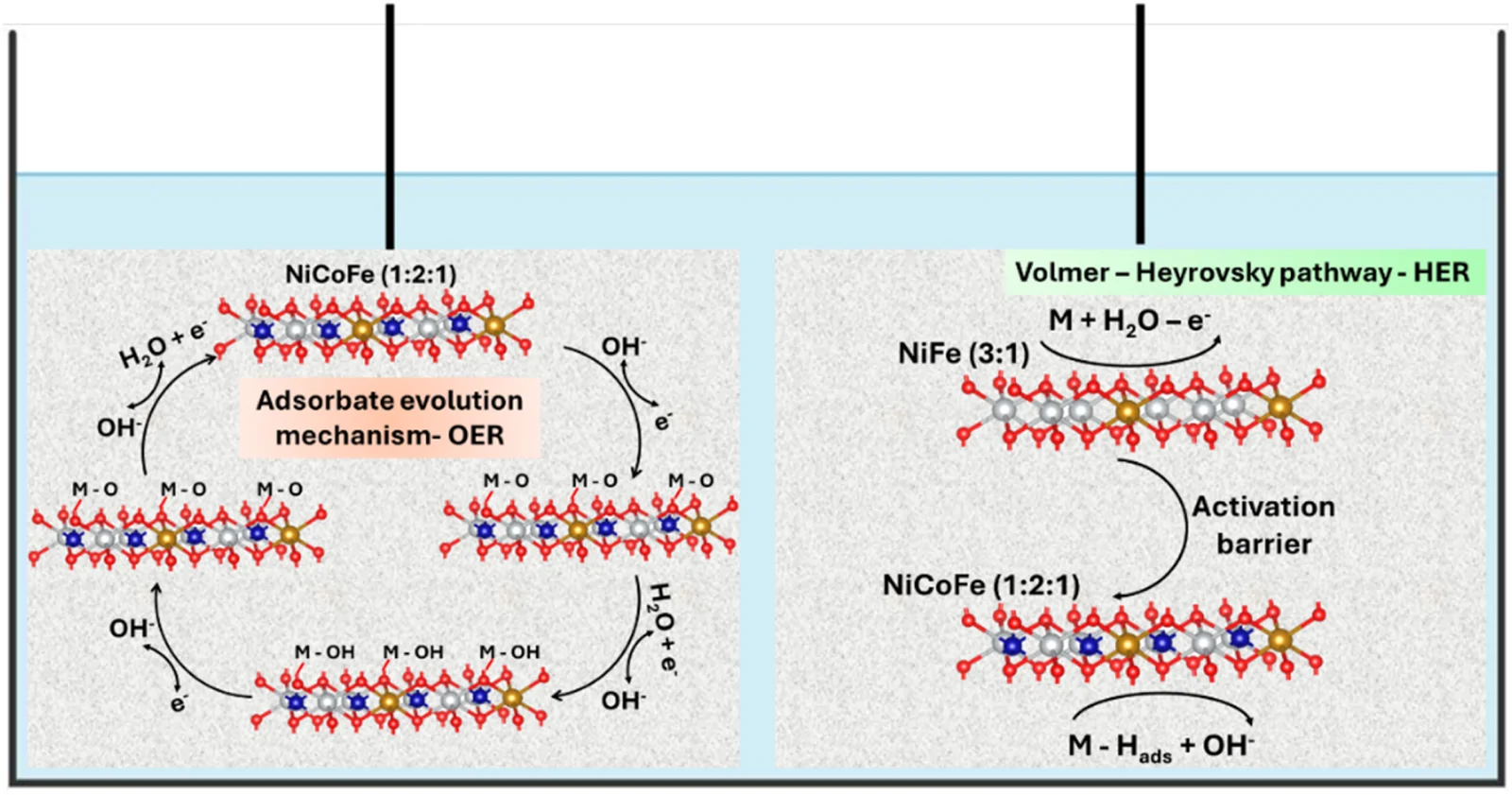
Illustration of plausible pathway and mechanism involved electrocatalytic overall water splitting.

### 3.5 Photo-electrochemical overall water splitting analysis

Fig. 9(a) and (c) shows the comparison of LSV curves of NiFe LDH and Co substituted NiFe LDH for OER and HER analysis, respectively, with their corresponding Tafel slope represented in Fig. 9(b) and (d). The photocurrent density of bare NiFe LDH is extremely low for the applied potential, reaching up to only 0.82 mA cm^−2^ at the highest applied potential of 2.1 V *vs.* RHE.

**Fig. 9:**
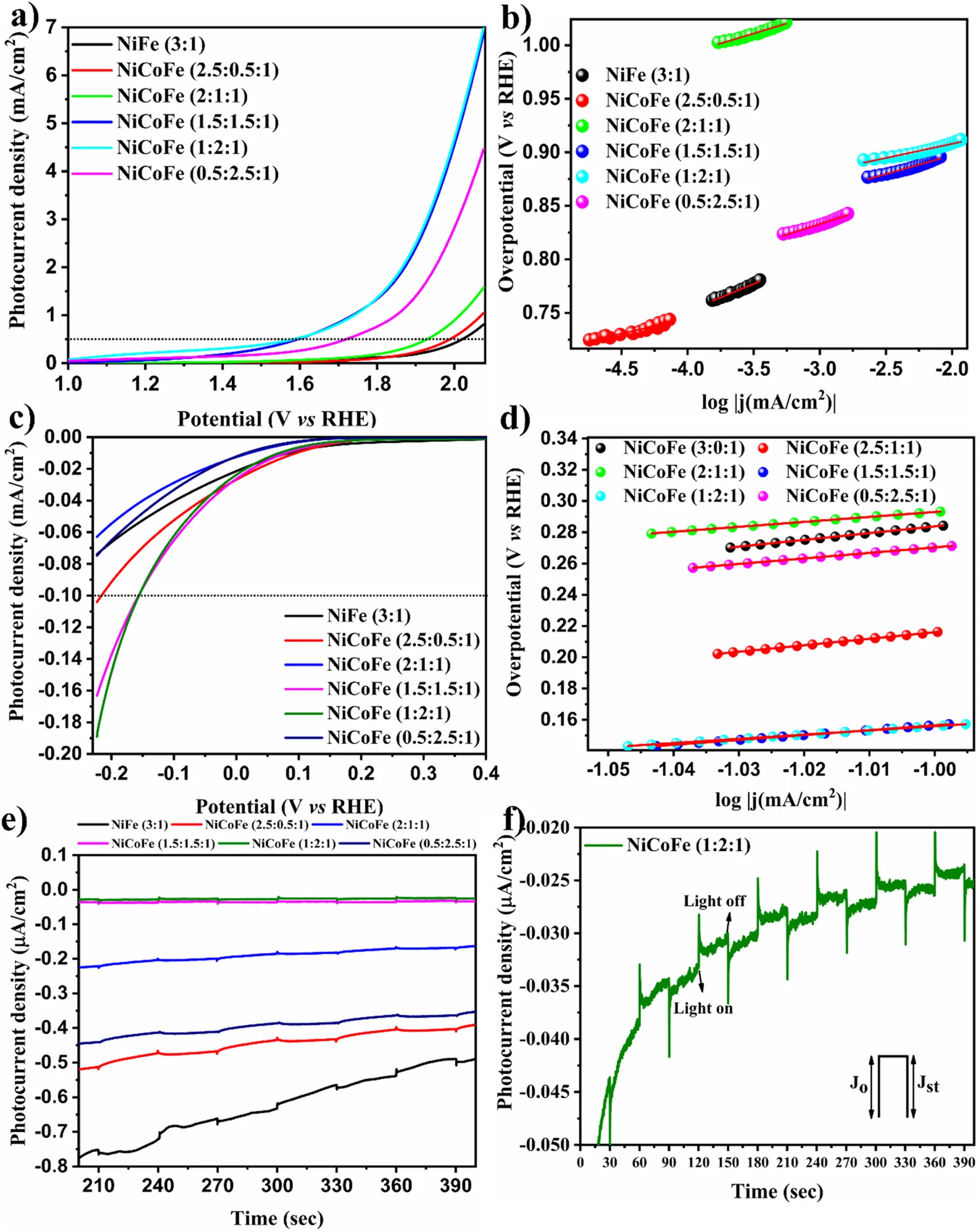
(a) Linear sweep voltammetry (LSV) curve for OER using NiFe and NiCoFe LDH @ 50 mV; (b) OER Tafel slope; (c) *i*–*t* chronopotentiometry analysis using NiFe and NiCoFe LDH @ 0 V; (d) linear sweep voltammetry (LSV) curve for HER using NiFe and NiCoFe LDH @ 50 mV; (e) HER Tafel slope; (f) *i*–*t* chronopotentiometry analysis using NiCoFe LDH (1 : 2 : 1).

However, for NiCoFe (1 : 2 : 1) composition, the photocurrent density reached up to 7 mA cm^−2^, which is 8.4 times higher than that of binary NiFe (3 : 1) LDH. Furthermore, at 0.5 mA cm^−2^, the lowest overpotential of 350 mV was estimated to be achieved by NiCoFe (1 : 2 : 1) composition with a lowest Tafel slope value of 26.4 mV dec^−1^. These extremely low kinetics indicate rapid charge-transfer kinetics, with the rate-determining step shifting from hydroxide adsorption to likely O–O bond formation or O_2_ adsorption. The pristine NiFe (3 : 1) has the Tafel value of 50 mV dec^−1^, suggesting that the formation of the N–OOH intermediates govern the OER kinetics. The shift in the reaction pathway without the intermediates directly to the O_2_ desorption step due to the Co substitution, forming a stronger hybridization and p–n heterojunction. Subsequently, the LSV curve of HER shows the photocurrent density reaching up to −0.19 mA cm^−2^ for NiCoFe (1 : 2 : 1), whereas for bare NiFe (3 : 1), the photocurrent density reaches only up to −0.075 mA cm^−2^, which is almost 2.5 times less than the optimal Co substituted NiCoFe photocatalyst. The overpotential values appear to be lowest for NiCoFe (1 : 2 : 1) and (1.5 : 1.5 : 1), at 155 mV and 156 mV at −0.1 mA cm^−2^, whereas the Tafel slopes were 270 mV dec^−1^ and 312 mV dec^−1^, respectively. This large slope value suggests the Volmer step involving water dissociation and formation of adsorbed hydrogen, M + H_2_O + e^−^ → M − H* + OH^−^. However, in comparison with bare NiFe (3 : 1), the overpotential doesn't reach 0.1 mA cm^−2^, and the Tafel slope value was much higher, up to 432 mV dec^−1^, thereby limiting the HER rate despite the photo-generated electrons.

The photocurrent response over time of the prepared photo-electrodes was tested using *i*–*t* chronopotentiometry at 0 V *vs.* Ag/AgCl under ON and OFF light conditions, as shown in Fig. 9(e) and (f). A higher photo-current density was exhibited by NiCoFe (1 : 2 : 1) followed by NiCoFe (1.5 : 1.5 : 1), which is consistent with the LSV curves. The trend in the photo-current density was found to be NiCoFe (1 : 2:1) > NiCoFe (1.5 : 1.5 : 1) > NiCoFe (2 : 1 : 1) > NiCoFe (0.5 : 2.5 : 1) > NiCoFe (2.5 : 0.5 : 1) > NiFe (3 : 1). This trend proves that cobalt inclusion has sought to reduce the recombination of photo-generated excitons by serving as the trapping centers. Similar observations have been previously reported by Duo Pan *et al.*, where NiCoFe mixed metal oxides with a similar ratio of 1 : 2 : 1 tend to provide a strong transient photoresponse.^31^ Ideally, before illumination, the concentration of holes remains constant and the current density measured at that point is *j*_0._ As the light is illuminated, the photocurrent increases, and the hole transfer process is excited (*k*_tr_), competing with recombination (*k*_rec_), and reaches a steady state or equilibrium where the hole concentration is constant. The photocurrent density at this steady state point is *j*_st_, and upon switching OFF the illumination, the current density falls, and the holes combine with the electrons, moving to the surface of the catalyst. This steady state current ratio is given by,^32,33^
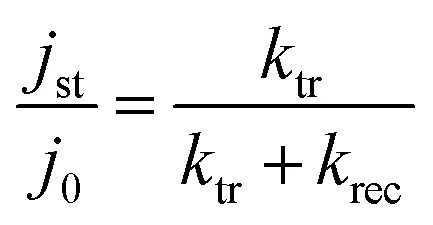


The *j*_st_/*j*_0_ values of NiFe (3 : 1) were found to be 1.13, and NiCoFe (1 : 2 : 1) was 1.54, implying that the rate of transfer of excitons is improved for optimal Co inclusion. This inference has been validated further by photoluminescence spectra and transient response photoluminescence spectra (TRPL), which will be discussed in the further sections. The metrics obtained from the photo-electrochemical analysis are tabulated in Table 4. Herein, the *j*_st_/*j*_0_ ratio together with the TRPL lifetimes (Table 6) are used only as qualitative, indirect representations for improved charge-transport/trapping behaviour, not as a mobility value, where optimal Co inclusion has suppressed the recombination rate of excitons.

**Table 4:** Photo-electrochemical overall water splitting metrics

Sample	OER		HER		Steady state current (*j*_st_/*j*_0_)
	Overpotential @ 0.5 mA cm^−2^–50 mV sec	Tafel slope (mV dec^−1^)	Overpotential @ −0.1 mA cm^−2^–50 mV sec	Tafel slope (mV dec^−1^)	
NiFe (3 : 1)	790 mV	50.83	—	432	1.13
NiCoFe (2.5 : 0.5 : 1)	750 mV	29.28	216 mV	415	1.11
NiCoFe (2 : 1 : 1)	690 mV	37.34	—	316	1.108
NiCoFe (1.5 : 1.5 : 1)	360 mV	34.98	156 mV	312	1.13
NiCoFe (1 : 2 : 1)	350 mV	26.4	155 mV	270	1.54
NiCoFe (0.5 : 2.5 : 1)	480 mV	39.21	—	353	1.07

The Nyquist plot attained from the photo-electrochemical impedance spectroscopy for the prepared NiFe and Co modified NiFe LDH is shown in Fig. S4(b). From the Nyquist plot profile, it can be observed that the binary LDH (NiFe-3 : 1) exhibits a higher charge transfer resistance (*R*_ct_) in the high-frequency region, whereas Co substitution significantly decreases *R*_ct_ by forming a minimal semicircle. The compositions with optimal Co substitution, NiCoFe (1 : 2 : 1) and NiCoFe (1.5 : 1.5 : 1), tend to exhibit minimal charge-transfer resistance at the electrode–electrolyte interface, thereby improving charge-transfer kinetics. These inferences are in complement with the overpotential and Tafel slope values attained from the overall photo-electric water splitting analysis.

### 3.6 Photocatalytic H_2_ evolution analysis *via* water splitting

The light-driven splitting of water was carried out using the prepared NiFe (3 : 1) and NiCoFe (1 : 2 : 1) nanostructures with TEOA as the sacrificial agent, and the amount of evolved H_2_ is shown in the Fig. 10(a) and (b). For the overall 3 hours illumination time, the ternary LDH NiCoFe (1 : 2 : 1) outperforms the bare NiFe (3 : 1) LDH by producing an average of 925 µmol h^−1^ g^−1^of H_2_, whereas the latter produces 752 µmol h^−1^ g^−1^ of H_2_. This improvement is attributed to the synergistic substitution of Co in Ni, sites, thereby enhancing charge transfer and catalytic activity. Furthermore, the electronic structure modulation induced by the hybridization of the Co inclusion facilitates efficient charge-carrier separation and transport. Furthermore, at an optimal Co concentration, a strong p–n heterojunction is formed, which further reduces the exciton recombination rate, thereby improving the lifetime of the charge carriers. Table 5 provides a broader comparison with the recent state of the art LDH – based catalysts for photocatalytic H_2_ evolution, and it is noteworthy that the reported simplistic electronic modification of optimal Co induced NiFe LDH catalyst demonstrates superior H_2_ evolution rate.

**Fig. 10:**
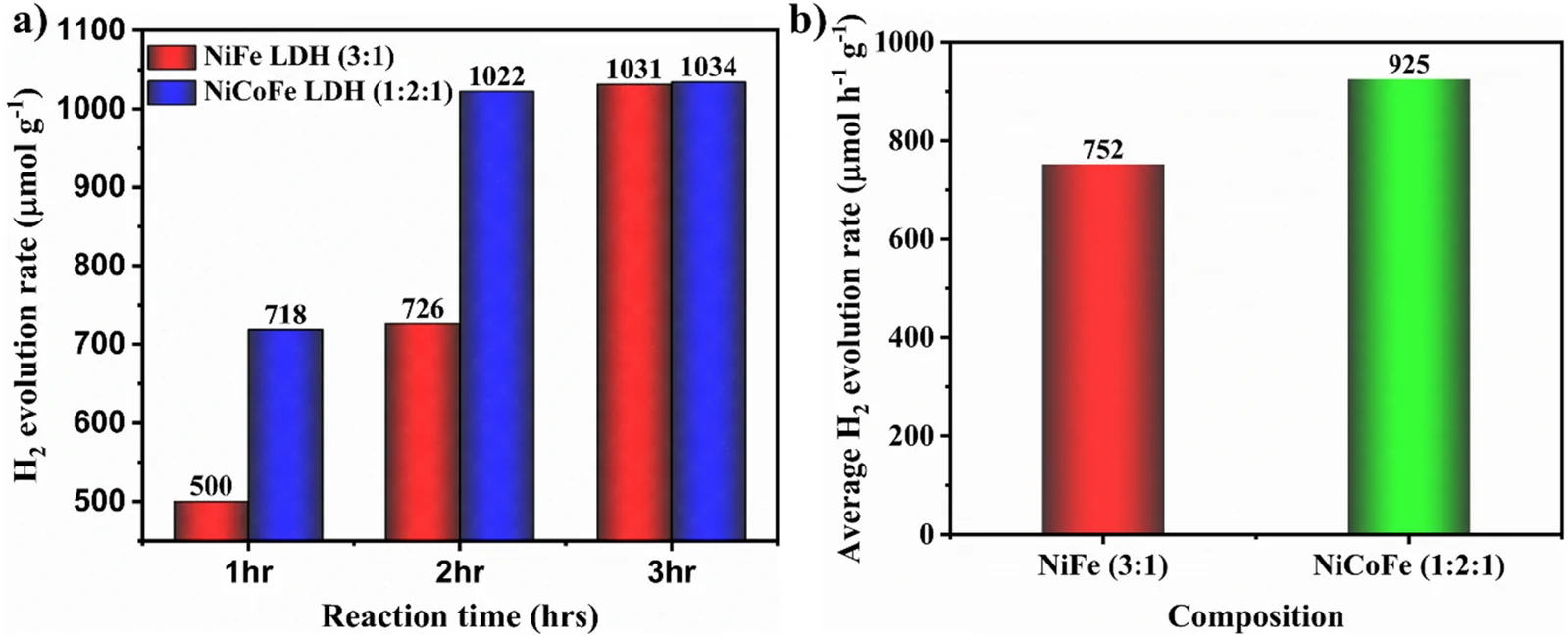
(a) Photocatalytic H_2_ evolution with TEOA as the sacrificial agent; (b) average H_2_ evolution rate using NiFe LDH and NiCoFe (1 : 2 : 1).

**Table 5:** Comparison of previously reported LDH based catalysts for H_2_ evolution

Catalyst	Light source	Condition	Productivity (µmol h^−1^ g^−1^)	Reference
CoAl LDH@g-C_3_N_4_	Sunlight	10 mg catalyst in 50 mL H_2_O + 10 vol% glycerol solution	430.7	34
CuAl-g-C_3_N_4_	LED lamp (445 nm)	0.5 g L^−1^ catalyst in 2 ml 10 vol% TEOA solution	54	35
Cu@NiFe LDH	100 W Hg lamp	50 mg catalyst in 300 ml (25 vol%) methanol solution	54.2	36
NiCoV LDH/g-C_3_N_4_	250 W Hg lamp	0.1 g catalyst on 100 ml (10 vol%) methanol solution	1673.4 µmol h^−1^g^−1^ (4 h reaction time)	37
CdS/NiCoAl LDH	300 W Xenon lamp (420 filter)	20 mg catalyst in 50 ml DI water containing Na_2_S (0.25 mol L^−1^) and Na_2_SO_3_ (0.35 mol L^−1^)	2116.1	38
NiAl LDH/CNTP-MOF	300 W Xenon lamp (UV cut off filter)	10 mg catalyst in 50 ml (10 vol% methanol) solution	382.8	39
NiCoFe (1 : 2 : 1)	500 W Tungsten lamp	10 mg catalyst in 50 ml (20 vol%) TEOA solution	925	This work

### 3.7 Steady state photoluminescence and time-resolved photoluminescence

Fig. 11(a) represents the PL steady state emission spectra and Fig. 11(b) of the prepared NiFe LDH and the varied composition of NiCoFe LDH samples. The PL emission spectra (Fig. 11(a)) carried out at the excitation wavelength of 360 nm in the emission band 400–680 nm, show emission peaks at 462 nm, 472 nm, and 482 nm, followed by a prominent emission peak at ∼650 nm. These peaks are attributed to radiative recombination of photoexcited electrons and holes. S. Nayak and K. Parida *et al.* reported a similar observation with NiFe LDH integrated with n-rGO and g-C_3_N_4_.^40^ This work reported on the intense emission band at 575 nm owing to the surface defect ascribed to the surface-trapped oxygen vacancies. Similarly, the strong emission band in the red region (∼650 nm) might be attributed to strong surface defects, and the quenched PL emission spectra correlate with the reduced recombination rate of the generated excitons.

**Fig. 11:**
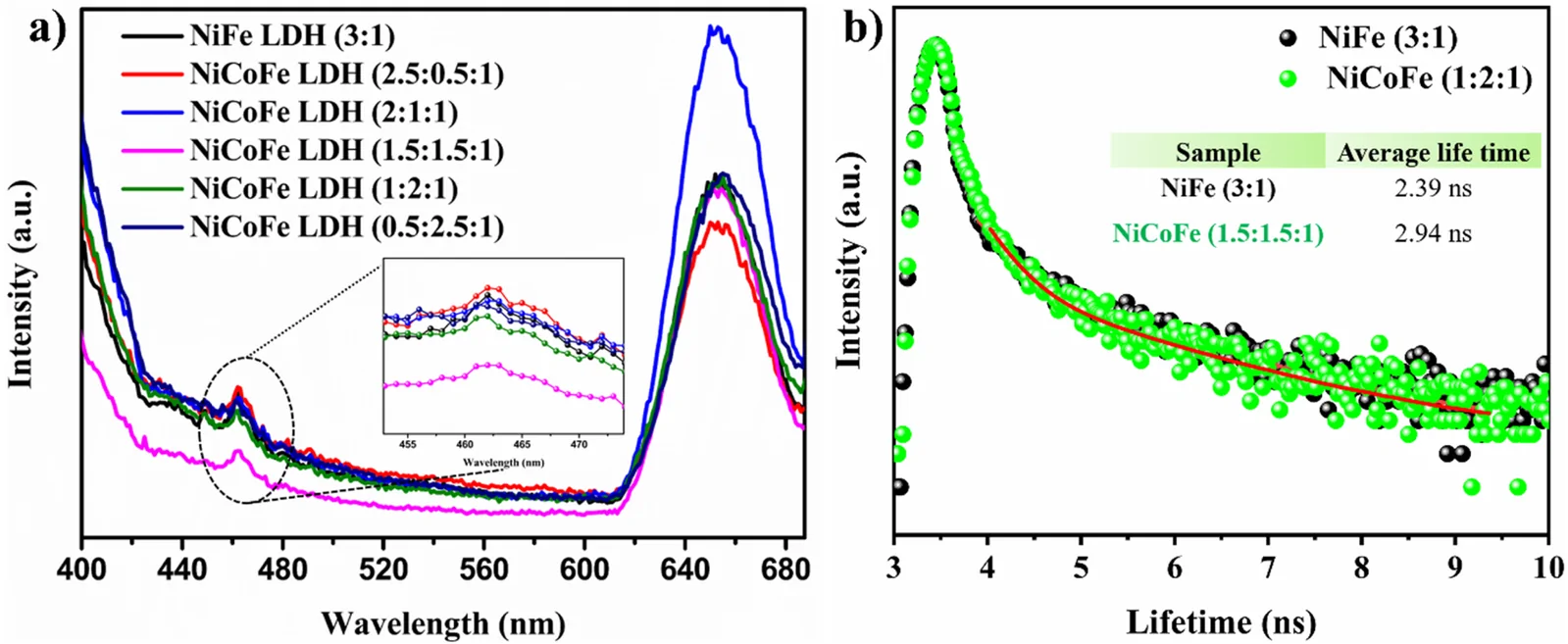
(a) Steady state photoluminescence spectra of NiFe (3 : 1) LDH and Co substituted NiFe LDH nanostructures; (b) lifetime decay curve of NiFe (3 : 1) and NiCoFe (1 : 2 : 1) sample.

Furthermore, the charge carrier dynamics and lifetime measurement were measured *via* TRPL at the emission wavelength of 460 nm, which resulted in the decay curves, and were fitted with the bi exponential function, the average lifetime time was measured using the following equation:*τ*_avg_ = Σ*A*_i_*τ*_i_^2^/*A*_i_*τ*_i_where *A*_i_ are the corresponding amplitudes, and *τ*_i_ are the fluorescent lifetime, the fitted values and the corresponding lifetime values are tabulated in Table 6. The average lifetime was found to be 2.39 ns for NiFe (3 : 1), and for NiCoFe (1 : 2 : 1) the lifetime of the charge carriers extended up to 2.94 ns. This indicates a prolonged carrier lifetime attributed to the superior hole-transfer and trapping capacities upon Co inclusion. Furthermore, the prolonged fluorescence lifetime can be related to the inhibited non-radiative transition after Co inclusion and the ligand to metal charge transfer transitions *via* the charge transfer process of O^2−^ → Ni^2+^, O^2−^ → Fe^3+^ and oxygen vacancies trapping the photo-excited electron from the conduction band of the prepared NiCoFe heterostructures.^41^ These act as intermediate states during the decay process, thereby responsible for the longer decay lifetime and efficient photocatalytic and photoelectrocatalytic activities.

**Table 6:** Weighing factors and time constant factors used for the bi-exponential fitting of the TRPL spectra and the deduced average lifetime

Sample	B1	B2	*τ*_1_	*τ*_2_	*χ* ^2^	Average lifetime
NiFe (3 : 1)	3.13 × 10^−2^	1.53 × 10^−3^	1.20 × 10^−9^	6.71 × 10^−9^	1.35	2.39 ns
NiCoFe (1 : 2 : 1)	2.09 × 10^−2^	2.14 × 10^−3^	1.22 × 10^−9^	6.22 × 10^−9^	0.98	2.94 ns

### 3.8 Chemical state analysis using X-ray photo-electron spectroscopy

The chemical bonding environment and plausible chemical interactions were analyzed using X-ray photoelectron spectroscopy (XPS). The survey spectra shown in the Fig. S5 indicates the presence of Ni, Fe, C, and O alone for bare NiFe (3 : 1) LDH. Furthermore, as Co ions were included in the NiFe LDH, the photoelectron peak corresponding to the Co binding energy indicates their presence in the NiFe LDH matrix. The oxidation states of the constituent elements were further analyzed by deconvoluting the high-resolution XPS peaks of each element using CasaXPS version 2.3.24PR1.0, and the deconvoluted spectra are shown in Fig. 12(a)–(d). Fig. 11(a) represents the high-resolution spectra of Ni 2p with their fitted spin–orbit doublets of Ni 2p_3/2_ and Ni 2p_1/2_. The peaks were deconvoluted into 4 components, confirming the presence of mixed-valence states of Ni^2+^ and Ni^3+^, which is characteristic of surface oxidation in LDH. The binding energy position of the Ni^3+^ peak, located at 857.19 eV for binary NiFe (3 : 1), shifts positively for Co substituted composition NiCoFe (1 : 2 : 1) to 857.43 eV. Subsequently, a similar positive shift in the binding energy was also observed for peaks representing Ni^2+^. Ideally, a decrease in the electron density of Ni ions causes this positive shift in the binding energy, suggesting that Ni acts as an electron donor in the ternary matrix.

**Fig. 12:**
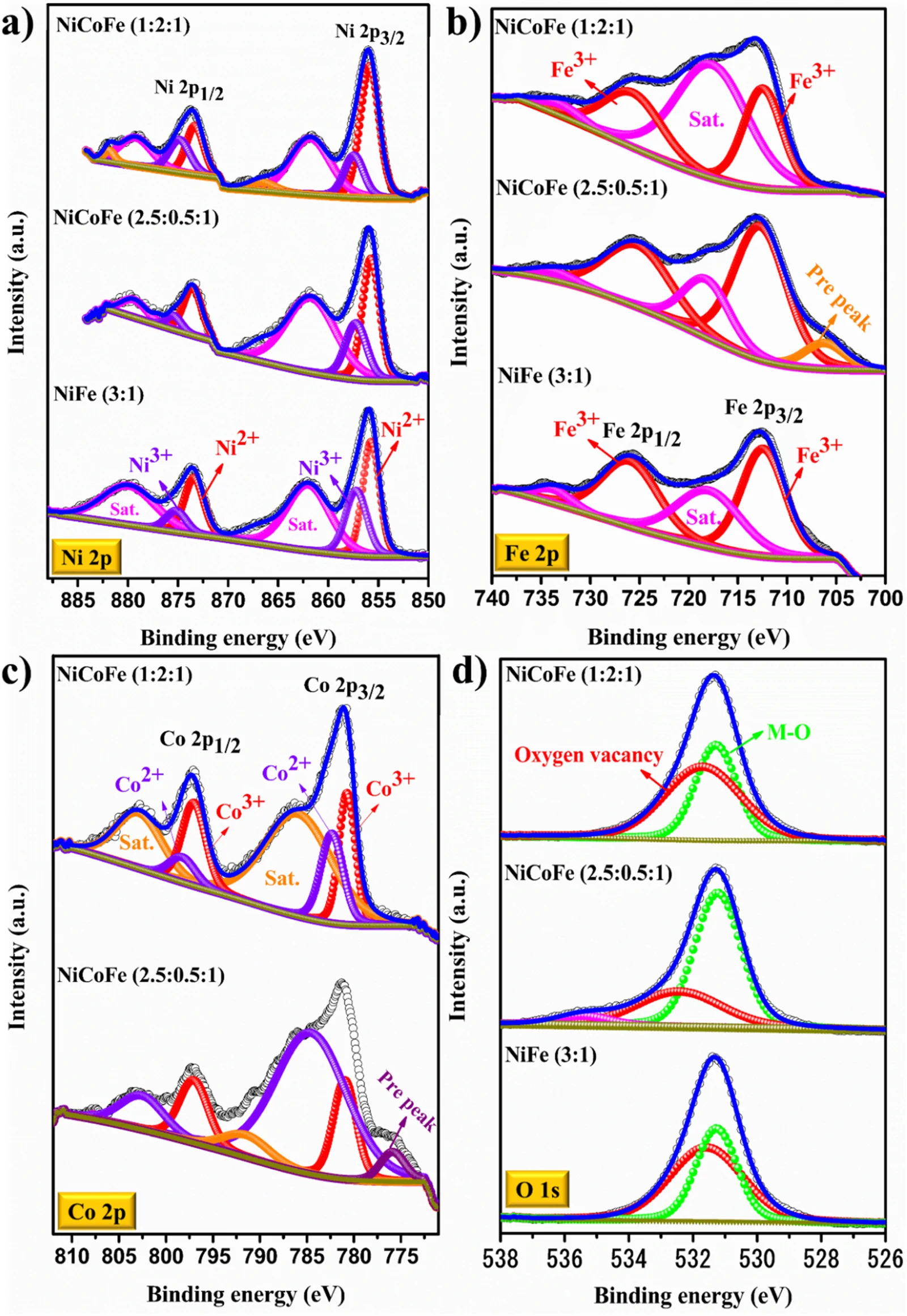
Deconvoluted high-resolution spectra of (a) Ni 2p; (b) Fe 2p; (c) Co 2p; (d) O 1s attained from bare NiFe (3 : 1) LDH, NiCoFe (2.5 : 0.5 : 1), and NiCoFe (1 : 2 : 1).

The high-resolution spectra of the Fe 2p peak and the deconvoluted spin–orbit doublets Fe 2p_3/2_ and Fe 2p_1/2_ are shown in Fig. 12(b), representing the existence of the Fe^3+^ ionic state. For the NiFe (3 : 1) sample, the peak at 712.41 eV appears to shift slightly positive to 712.45 eV upon Co substitution in NiCoFe (1 : 2 : 1). Similar to nickel, this positive shift in the binding energy implies a partial electron loss of electron density, indicating electrons are being drawn away from Fe sites and newly introduced to Co sites.

The high-resolution spectra of Co 2p spectra and their deconvolution of spin–orbit coupling corresponding to Co 2p_3/2_ and Co 2p_1/2_ are shown in Fig. 12(c). The peaks deconvolute into 4 peaks corresponding to the existence of Co^2+^ and Co^3+^ mixed oxidation states, which also corresponds to the previous reported work by Yanping Lin *et al.*^42^ Herein, as the Co ratio increases, the binding energy corresponding to Co^3+^ of NiCoFe (2.5 : 0.5 : 1) present in 784.71 eV shifts negatively to 782.32 eV for NiCoFe (1 : 2 : 1) composition. This negative shift corresponds to the increase in the electron density of cobalt atoms. The shift in the binding energies of the elements indicates a strong chemical interaction, and the positive shifts in the binding energies of Ni 2p and Fe 2p, coupled with a negative shift in the Co 2p binding energies, directly indicate the electron transfer from Ni and Fe sites to Co sites. This charge redistribution modulates the local electronic environment and shifts the d-band center of the active sites. The detailed peak lists attained from the deconvolution of high-resolution spectra are shown in Table 7.

**Table 7:** Binding energy position of deconvoluted high-resolution spectra of Ni, Fe, Co, and O

Peaks		Peak binding energy (eV)		
		NiFe (3 : 1)	NiCoFe (2.5 : 0.5 : 1)	NiCoFe (1 : 2 : 1)
Ni 2p	Ni^2+^	855.71	855.72	855.86
Ni 2p_3/2_		873.39	873.54	873.29
Ni 2p_1/2_	Ni^3+^	857.19	857.21	857.42
		875.17	875.40	874.83
Fe 2p	Fe^3+^	712.41	712.70	712.45
Fe 2p_3/2_		725.98	725.17	725.66
Fe 2p_1/2_				
Co 2p	Co^2+^	—	781.02	780.72
Co 2p_3/2_		—	797.06	797.01
Co 2p_1/2_	Co^3+^	—	784.71	782.32
		—	803.34	798.26
O 1s	O 1s	531.58	531.22	531.69
		531.26	532.42	531.28
			535.42	

The O1 high-resolution spectra and its deconvolution are shown in Fig. 12(d). The bare NiFe (3 : 1) deconvolutes into multiple peaks at 531.58 and 531.^26^ corresponding to surface hydroxyl (OH^−^) groups and metal–oxygen (M–O) lattice groups of LDH layers. Furthermore, NiCoFe (2.5 : 0.5 : 1) appears to exhibit a broader distribution of oxygen species at 531.22 eV and 532. 42 eV, and 535.42 eV corresponding to lattice oxygen, highly oxidative surface oxygen species, and adsorbed water, respectively. However, for the NiCoFe (1 : 2 : 1) composition, the peaks shift towards higher binding energies of 531.69 eV and 532.28 eV, indicating a stronger M–O network and an increased concentration of oxygen vacancies. Also, by comparing the area under the curve, NiCoFe (1 : 2 : 1) tends to have a higher area under the oxygen vacancy curve of about 2,13,523 in comparison with bare NiFe (169 690). However, a massive peak shift has been observed for NiCoFe (2.5 : 0.5 : 1), indicating a massive presence of chemisorbed water and surface carbonates. So, these layers, instead of facilitating the reaction, likely hinder access to the active metal sites, acting as a physical barrier to charge carriers. The electrocatalytic and photo-electrocatalytic results also tend to provide a superior performance for NiCoFe (1 : 2 : 1), confirming the inference.

### 3.9 Electron paramagnetic resonance spectroscopy

The electron paramagnetic resonance (EPR) spectroscopy was employed to investigate the influence of Co substitution on the local electronic and magnetic environment of NiFe LDH (3 : 1), and the attained spectra is shown in Fig. S10. The attained spectra is similar to that of as reported by K. K. Mandari *et al.*, where herein, the pristine NiFe (3 : 1), and NiCoFe (1 : 2 : 1) exhibit broad resonance features centred at 348 mT, and 335 mT, respectively.^43^ This shift in magnetic field towards lower resonance magnetic field following Co incorporation corresponds to the Co incorporation modifying the local paramagnetic environment and magnetic environment within the LDH framework. Furthermore, the comparatively weaker response of NiCoFe (1 : 2 : 1) accompanied by changes in the spectral profile, may reflect in exchange coupling, spin-relaxation behaviour, and metal–oxygen coordination. Subsequently, the *g*-factor was determines using *g* = *hν*/*µ*_B_*B*_res_, where *h* = 6.626 × 10^−34^ J s^−1^, *ν* = 9.4 × 10^9^ Hz, and *µ*_B_ = 9.274 × 10^−24^ J T^−1^. From this relation, the *g*-value was derived to be 1.930, and 2.003 for NiFe (3 : 1), and NiCoFe (1 : 2 : 1) respectively. The *g* – values between 2.00 to 2.03 correspond commonly to the localized unpaired electrons, including defect-related paramagnetic centres.

The reduced EPR amplitude after Co substitution may associate from the alterations in spin–spin exchange interactions, local coordination symmetry, and spin-relaxation dynamics, rather than merely from variations in defect concentration. The detected alterations in resonance position and spectrum intensity indicate that Co substitution affects the local electronic configuration of NiFe LDH *via* interactions among Ni, Co, Fe, and their adjacent oxygen-containing ligands. This explanation aligns with the electrical structural variation shown by the valence-band XPS and Mott–Schottky assessments. The notable cathodic shift in the observed flat-band potential from – 0.62 V for NiFe (3 : 1) to – 0.87 V *vs.* Ag/AgCl for NiCoFe (1 : 2 : 1) highlights the alteration of interfacial electronic characteristics following Co inclusion. These modifications might influence the modified charge-transfer properties and photoelectrochemical performance of the Co-substituted NiFe LDH catalyst.^43^

### 3.10 Plausible mechanism in photo-electrocatalysis and photocatalysis

Under light irradiation, the synergetic metallic ions dictate superior photon capture and exciton management. The strong hybridization between Ni 3d, Fe 3d, and Co 3d orbitals effectively narrows the optical bandgap to 1.91 eV and upshifts the VBM closer to the Fermi level. This not only improves the light-harvesting capability but also facilitates faster hole-transfer kinetics to the catalyst–electrolyte interface.^44^

Besides, this hybridization initiates the formation of a plausible intrinsic p–n heterojunction, as evidenced by the inverted V-shaped transition in the Mott–Schottky profile, shift in binding energies in XPS chemical environment and shift in EPR spectra as well. This heterojunction generates a strong built-in electric field that serves as a primary driving force for the rapid spatial separation of photo-generated electron–hole pairs. When illuminated, the photogenerated electrons in the conduction band suppress recombination by being effectively captured by abundant surface oxygen vacancies and discrete Co induced trapping centers. As observed from the TRPL, the extended lifetime of up to 2.94 ns confirms that the trapping mechanism, working in tandem with ligand-to - metal charge transfer transitions (O^2−^ → Ni^2+^ and O^2−^ → Fe^3+^), strongly inhibits the non-radiative recombination pathways. In due course, the spatially isolated, long-lived holes participate in the expedited OER, while the trapped electrons effectively reduce electrons for steady H_2_ evolution, thereby culminating in a highly stable, multifunctional catalyst architecture. The plausible heterostructure mechanism involved is shown in Fig. 13.

**Fig. 13:**
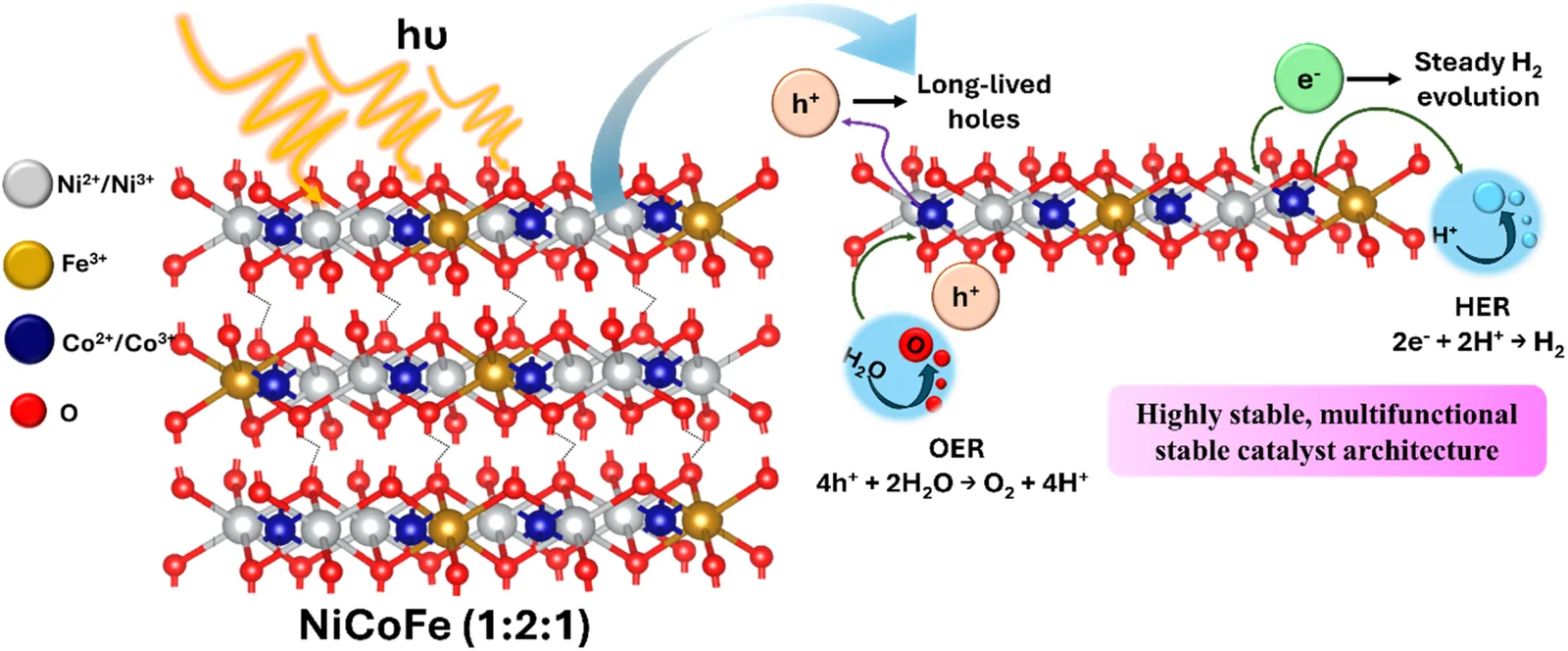
Illustration of a light-driven photocatalytic system involved in the dissociation of water.

## 4 Summary and conclusion

In summary, a series of ternary NiCoFe LDH nanostructures was strategically designed and synthesized *via* a facile hydrothermal approach to serve as highly efficient, multifunctional catalysts for water splitting. The systematic substitution of cobalt into the bare NiFe lattice triggered microstructural and electronic modulations. Morphologically, the optimal Co incorporation in the NiCoFe (1 : 2 : 1) composition prevented massive particle agglomeration, resulting in optimized 2D nanostructures that exposed a significantly higher density of catalytically active metal sites, as evidenced by their superior electrochemically active surface area. Optoelectronically, stronger hybridization of the Ni 3d, Co 3d, and Fe 3d orbitals narrows the optimal band gap and shifts the VBM closer to the Fermi level, thereby improving rapid hole-transfer kinetics. The band-edge potential analysis from the MS plot confirmed the optimal VBM and CBM potentials suitable for effective OER and HER processes and furthermore confirmed the p–n heterojunction like formation within the NiCoFe (1 : 2 : 1) matrix. The built-in electric field combined with surface oxygen vacancies, and optimized d-band centers confirmed from XPS, effectively tapped photo-excited electrons and reduced exciton recombination, thereby extending the lifetime to 2.94 ns.

These synergistic physical and electronic enhancements translated into outstanding multifunctional catalytic performance. As a bifunctional electrocatalyst, the NiCoFe (1 : 2 : 1) required ultra-low overpotentials of 50 mV for OER and 234 mV for HER. Under light irradiation, the PEC performance was markedly superior, yielding a photocurrent density of 7 mA cm^−2^ (8.4 times higher than that of pristine NiFe LDH) and a lower OER Tafel slope of 26.4 mV dec^−1^, indicating a rapid, direct oxygen desorption pathway. Furthermore, the direct photocatalytic activity demonstrated an average H_2_ evolution rate of 925 µmol h^−1^ g^−1^. Finally, this work elucidates the mechanism by which targeted cationic substitution fine-tunes the electronic band offsets and surface defect chemistry in layered transition-metal hydroxides, paving the way for a robust and cost-effective pathway toward scalable green hydrogen production technologies.

## Author contributions

Annamalai Kamalakkannan – conceptualization, data curation, formal analysis, investigation, methodology, visualization, writing – original draft, writing – review and editing; Salma Machiche – conceptualization, data curation; Balakumar Subramanian – resources; Sasirekha N. R. – resources; Mohamed Essalhi – conceptualization, and Otman Abida – conceptualization, resources, project administration, writing – review and editing, supervision, validation.

## Conflicts of interest

The authors declare that they have no known competing financial interests or personal relationships that could have appeared to influence the work reported in this paper.

## Supplementary Material

RA-OLF-D6RA08160F-s001

## Data Availability

The data supporting this article have been included as part of the supplementary information (SI). Supplementary information: additional structural, morphological, optical, electronic, electrochemical, photoelectrochemical, and stability characterization data, including EDX, DLS, UV–Vis DRS, Mott–Schottky, EIS, XPS, FESEM, XRD, EPR, and ICP–MS analyses. See DOI: https://doi.org/10.1039/d6ra08160f.
