# Supplementary material for: Cobalt induced electronic and defect structure modulation in nickel iron layered double hydroxides for electrochemical, photo-electrochemical and photocatalytic water splitting
Source: RSC Adv. 2026 Oct 27;16(49):66724–43. doi: 10.1039/d6ra08160f (PMC13711511; doi:10.1039/d6ra08160f)
Supplement: Supplementary file 1 [file RA-016-D6RA08160F-s001.pdf]

Supplementary Information (SI) for RSC Advances.
This journal is © The Royal Society of Chemistry

SUPPORTING INFORMATION

Cobalt Induced Electronic and Defect Structure Modulation in Nickel Iron Layered Double Hydroxides for Electrochemical, Photo-electrochemical and Photocatalytic Water Splitting

Annamalai Kamalakkannan[1*], Salma Machiche[1], Balakumar Subramanian[2], Sasirekha N R[3], Mohamed Essalhi[1] and Otman Abida[1*]

[1] *African Sustainable Agriculture Research Institute (ASARI), College of Agriculture and Environmental Sciences (CAES), Mohammed VI Polytechnic University (UM6P), Laayoune, Morocco.*

[2] *National Centre for Nanoscience and Nanotechnology, University of Madras, Chennai, India.*

[3] *Catalysis Laboratory, Centre of Advanced Study in Crystallography & Biophysics, University of Madras, Chennai, India.*

**** Corresponding author -*** *otman.abida@um6p.ma, and annamalai.kamalakkannan-ext@um6p.ma*

Table S1. Nomenclature table

Symbol	Description	Unit
η	Overpotential	mV
j	Current density	mA cm^{-2}
C_{dl}	Double-layer capacitance	mF cm^{-2}
ECSA	Electrochemically active surface area	Cm^2
E_g	Band gap energy	eV
E_{CB}	Conduction band potential	V
E_{VB}	Valence band potential	V
V_{fb}	Flat band potential	V
ND	Charge carrier density	cm^{-3}
τ	Charge carrier lifetime	ns
I	Current	A
F	Faraday constant (96485)	C mol^{-1}
R_{ct}	Charge transfer resistance	Ω
Rs	Solution resistance	Ω
hν	Photon energy	eV
α	Absorption coefficient	-
TOF	Turnover frequency	s^{-1}

Table S2. *Abbreviation table*

Abbreviation	Description
LDH	Layered Double Hydroxide
NiFe LDH	Nickel Iron layered double hydroxide
NiCoFe LDH	Nickel Cobalt Iron layered double hydroxide
HER	Hydrogen evolution reaction
OER	Oxygen evolution reaction
PEC	Photoelectrocatalysis
PL	Photoluminescence
TRPL	Time-resolved photoluminescence
LSV	Linear sweep voltammetry
EIS	Electrochemical impedance spectroscopy
XPS	X-ray photoelectron spectroscopy
XRD	X-ray diffraction
UV–Vis DRS	UV - Visible diffuse reflectance spectroscopy
MS	Mott - Schottky analysis
VB	Valence band
CB	Conduction band
VBM	Valence band maximum
CBM	Conduction band minimum

TEOA	Triethanolamine
EPR	Electron Paramagnetic Resonance

Table S3. *Concentrations of precursor salts used.*

**Composition**	**$NiCl_2.6H_2O$**	**$FeCl_2.6H_2O$**	**$CoCl_2.6H_2O$**
NiFe (3:1)	0.2674g	0.1014 g	--
NiCoFe (2.5:0.5:1)	0.2229g	0.1014 g	0.0447g
NiCoFe (2:1:1)	0.1783g	0.1014 g	0.0892g
NiCoFe (1.5:1.5:1)	0.1338g	0.1014 g	0.1340g
NiCoFe (1:2:1)	0.0891g	0.1014 g	0.1784g
NiCoFe (0.5:2.5:1)	0.0446g	0.1014 g	0.2235g

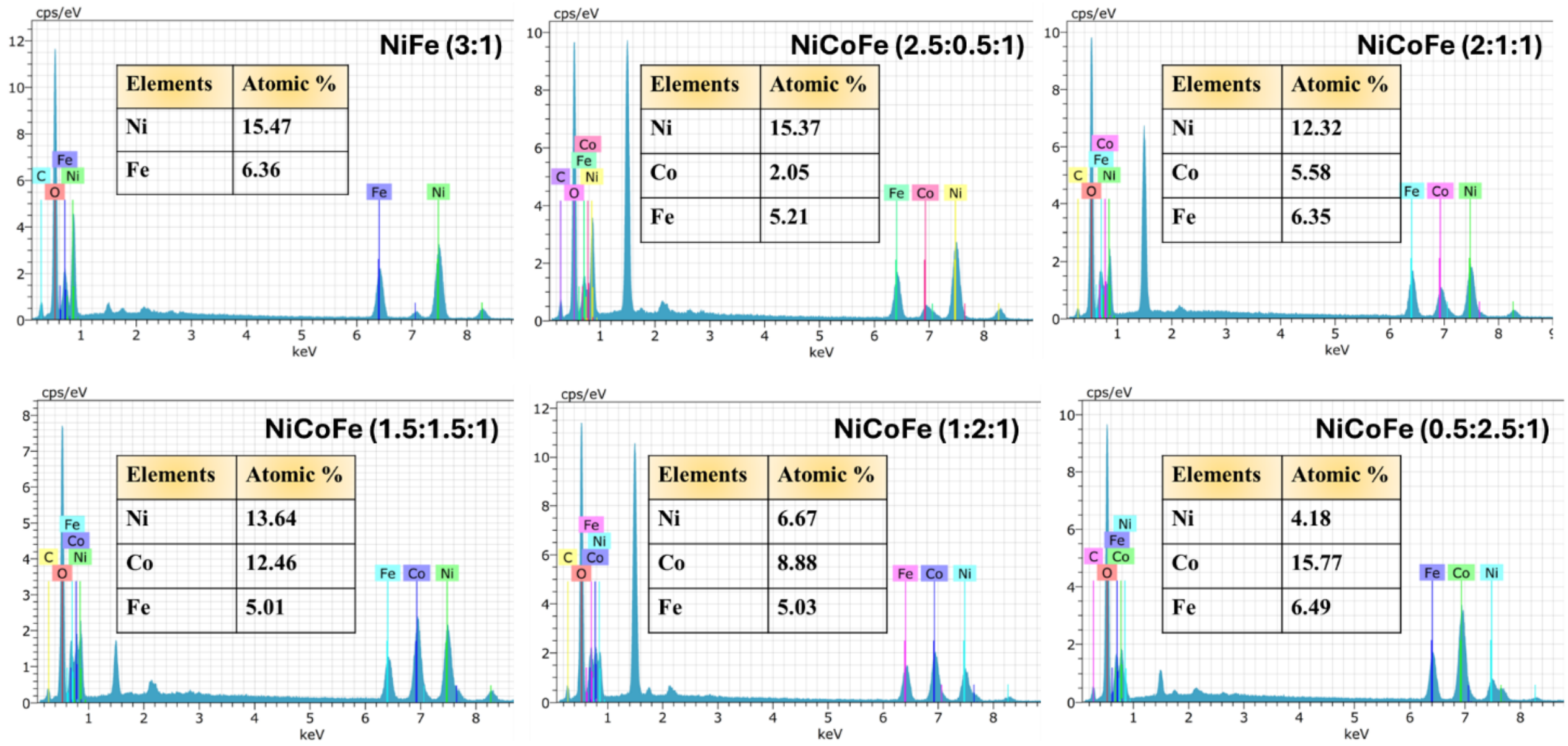

Figure S1. EDX spectrum of NiFe (3:1) LDH and Co substituted NiFe LDH nanostructures

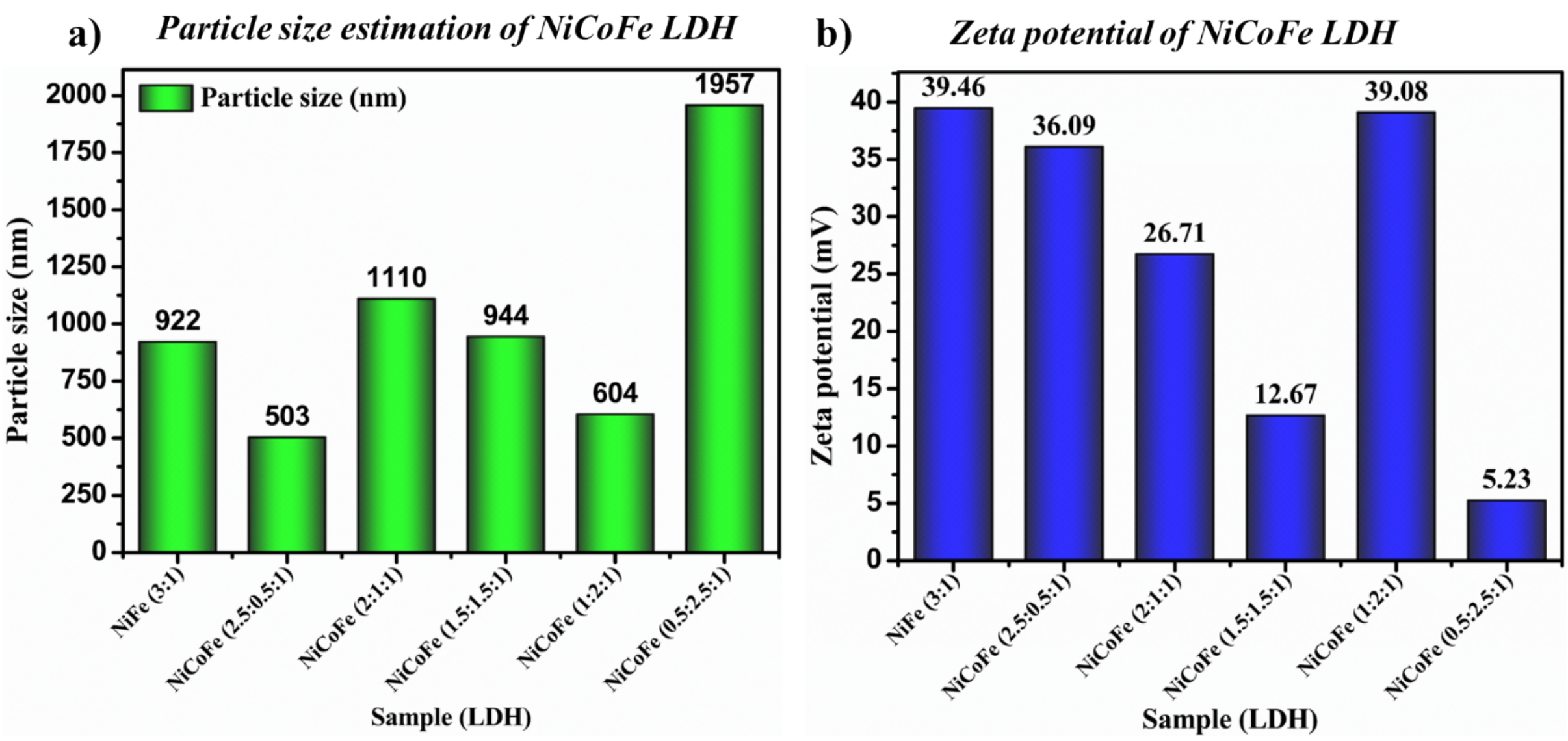

Figure S2. (a) Particle size estimation using DLS; (b) Surface potential analysis from DLS.

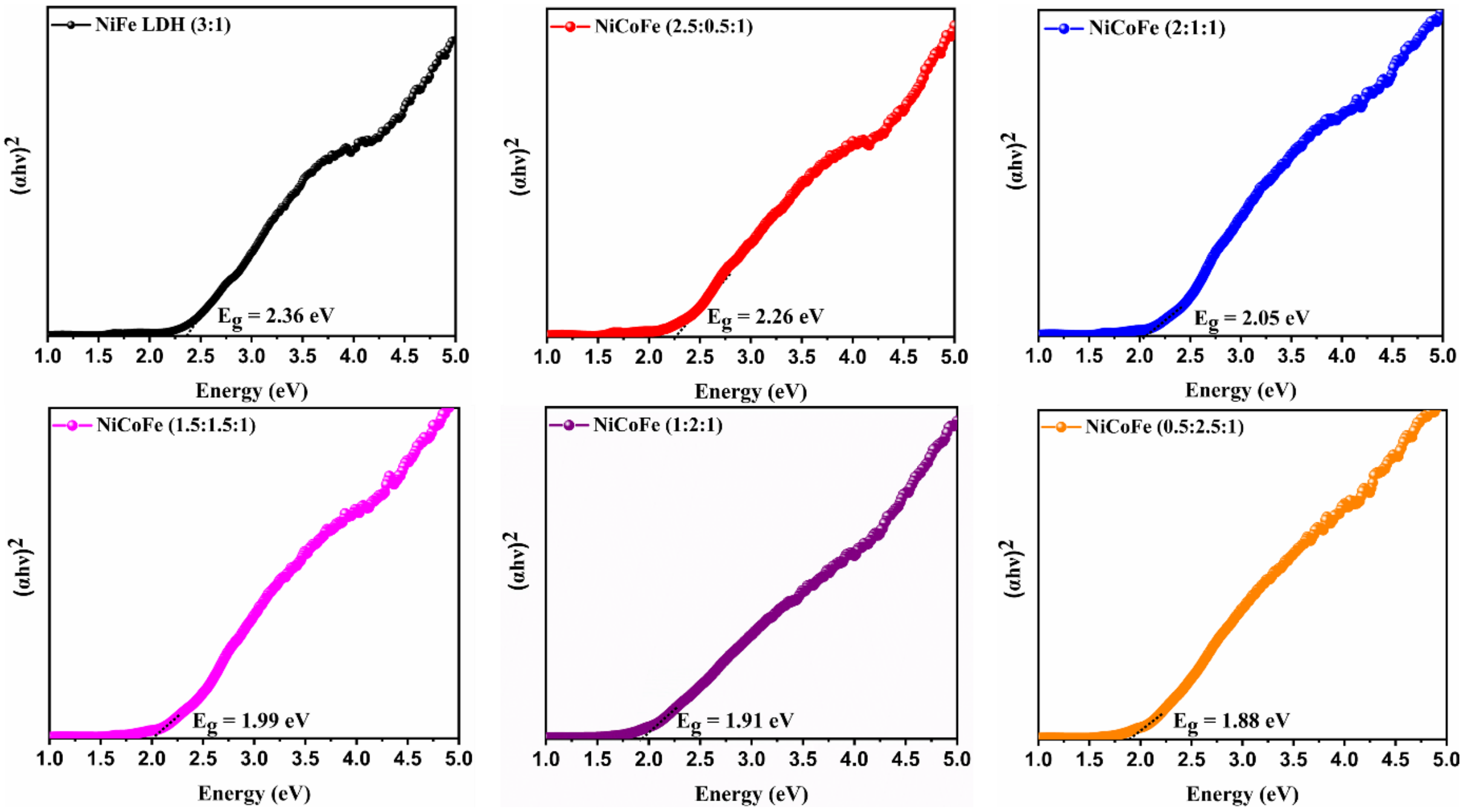

Figure S3: Tauc plot of NiFe (3:1) LDH and Co substituted NiFe LDH nanostructures

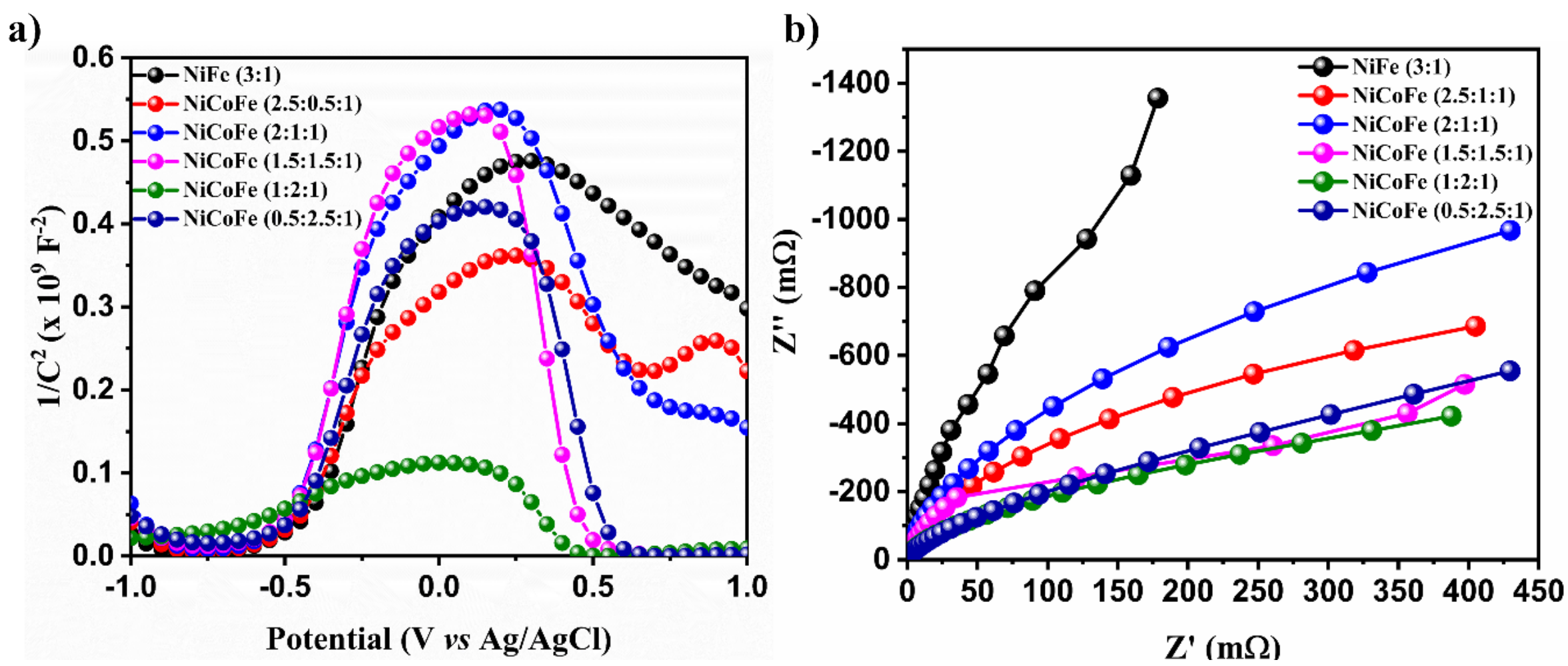

Figure S4: ***(a)*** *MS plot profile of NiFe (3:1) and Co substituted NiFe LDH heterostructures;* ***(b)*** *Photo-electrochemical Nyquist plot*

Table S4: Metrics from the fitted circuit of Nyquist plot from electrochemical overall water splitting analysis

Composition	R_s (Ω)	R_p (Ω)
NiFe (3:1)	1.35	3557
NiCoFe (2.5:0.5:1)	1.77	4029
NiCoFe (2:1:1)	1.13	4312
NiCoFe (1.5:1.5:1)	1.72	3546
NiCoFe (1:2:1)	1.20	3824
NiCoFe (0.5:2.5:1)	1.48	11787

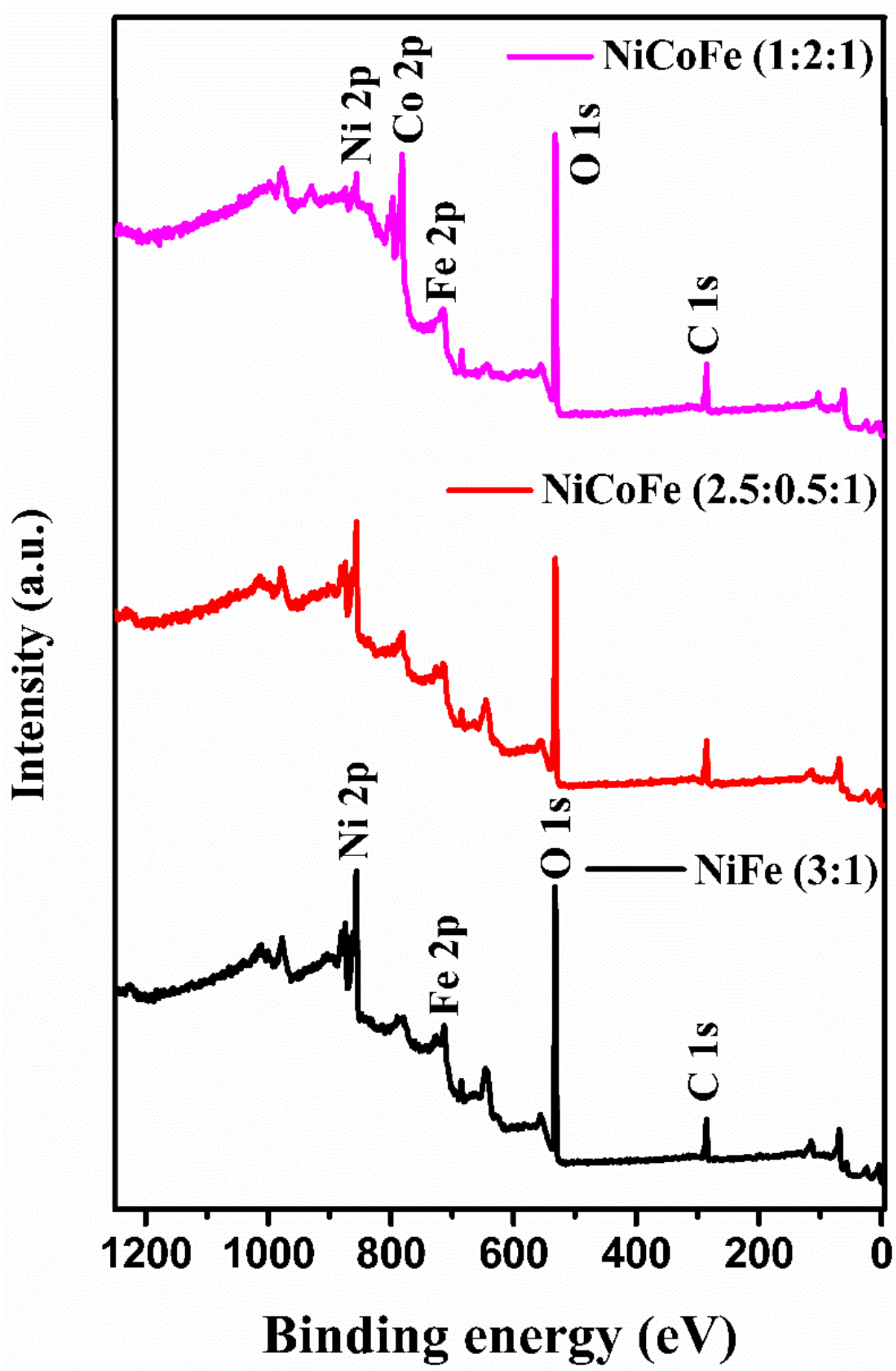

Figure S5. XPS survey spectra of NiFe (3:1), NiCoFe (2.5:0.5:1), and NiCoFe (1:2:1)

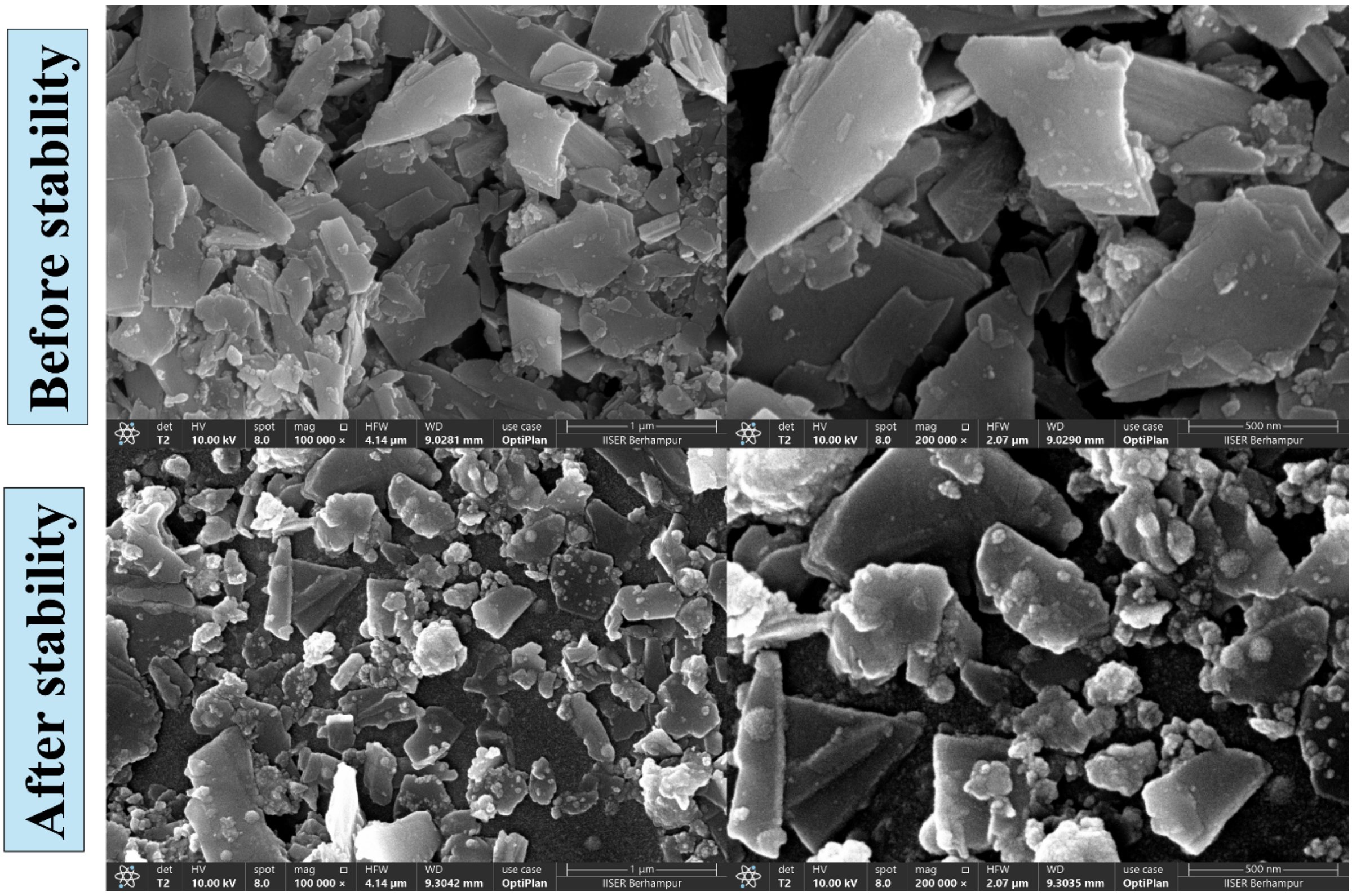

Figure S6. *FE-SEM micrographs of NiCoFe (1:2:1) catalyst coated on nickel foam before and after stability analysis @ 50 mA/cm²*

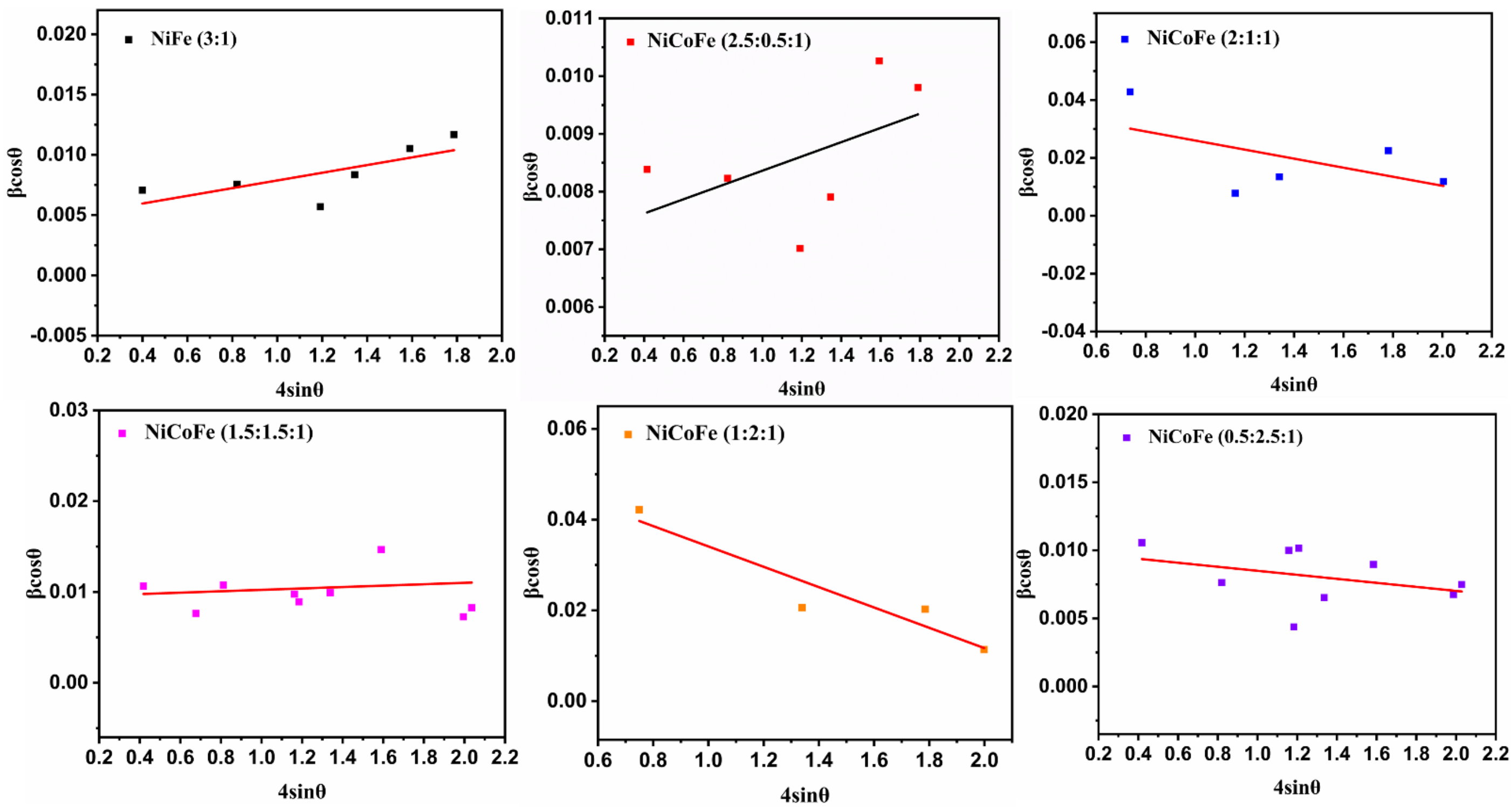

Figure S7. *Williamson – Hall plots of NiFe LDH, and Co substituted NiFe LDH heterostructures*

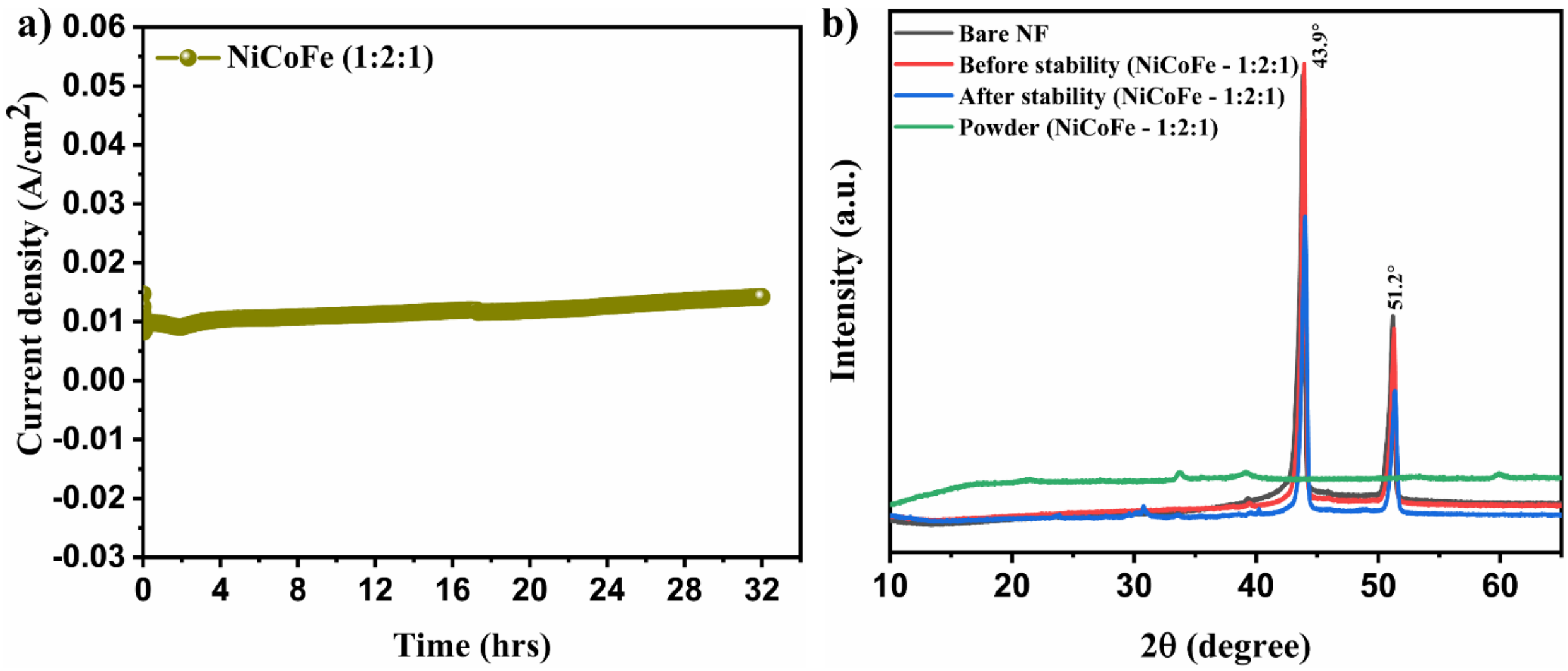

Figure S8. ***(a)*** *Stability of NiCoFe (1:2:1) to 32h h at 0.5V vs Ag/AgCl and* ***(b)*** *added post-stability XRD*

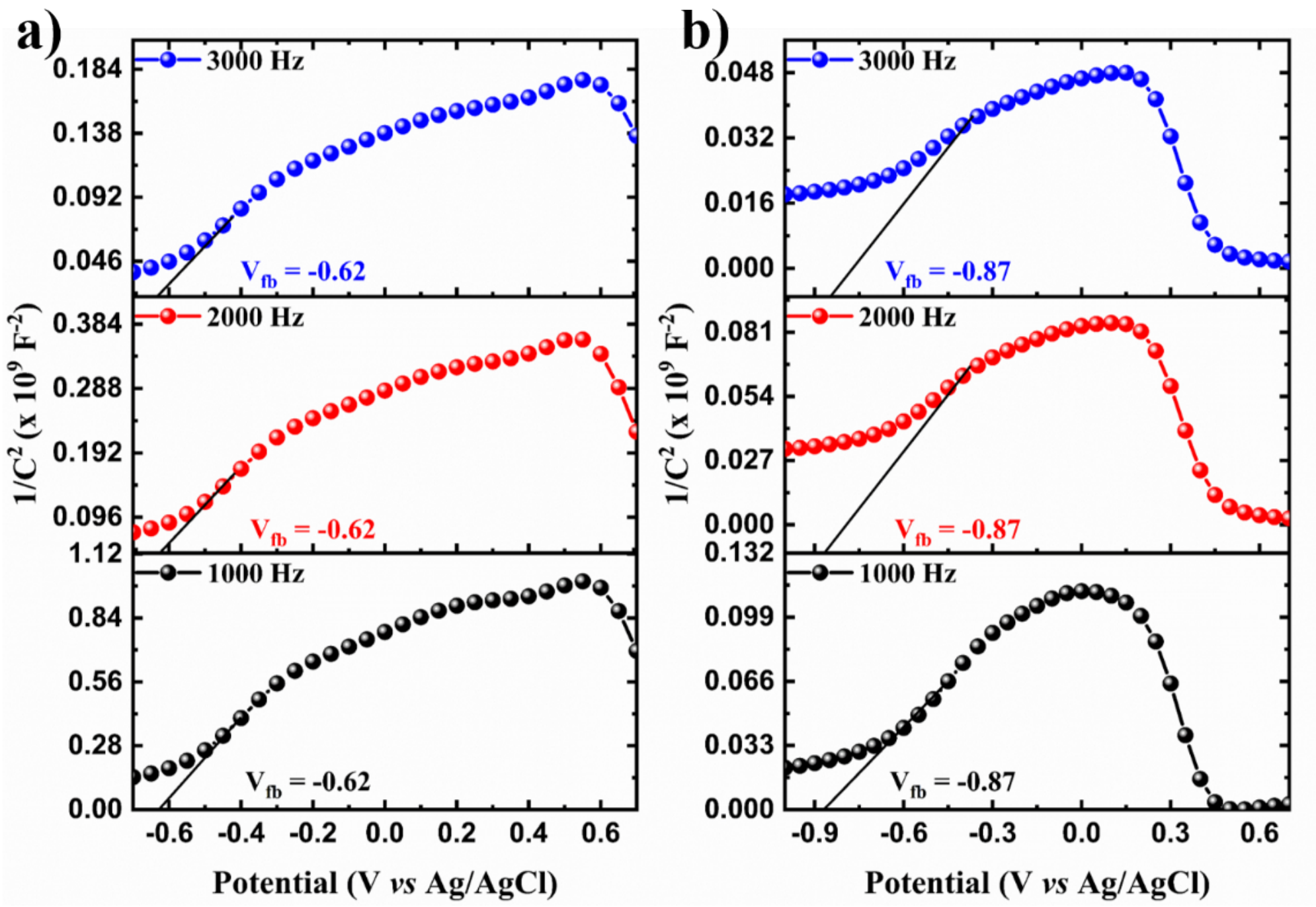

Figure S9. Frequency - dependent Mott–Schottky plots of ***(a)*** *pristine NiFe (3:1) LDH and* ***(b)*** *Co-substituted NiCoFe (1:2:1) LDH recorded at 1000, 2000, and 3000 Hz.*

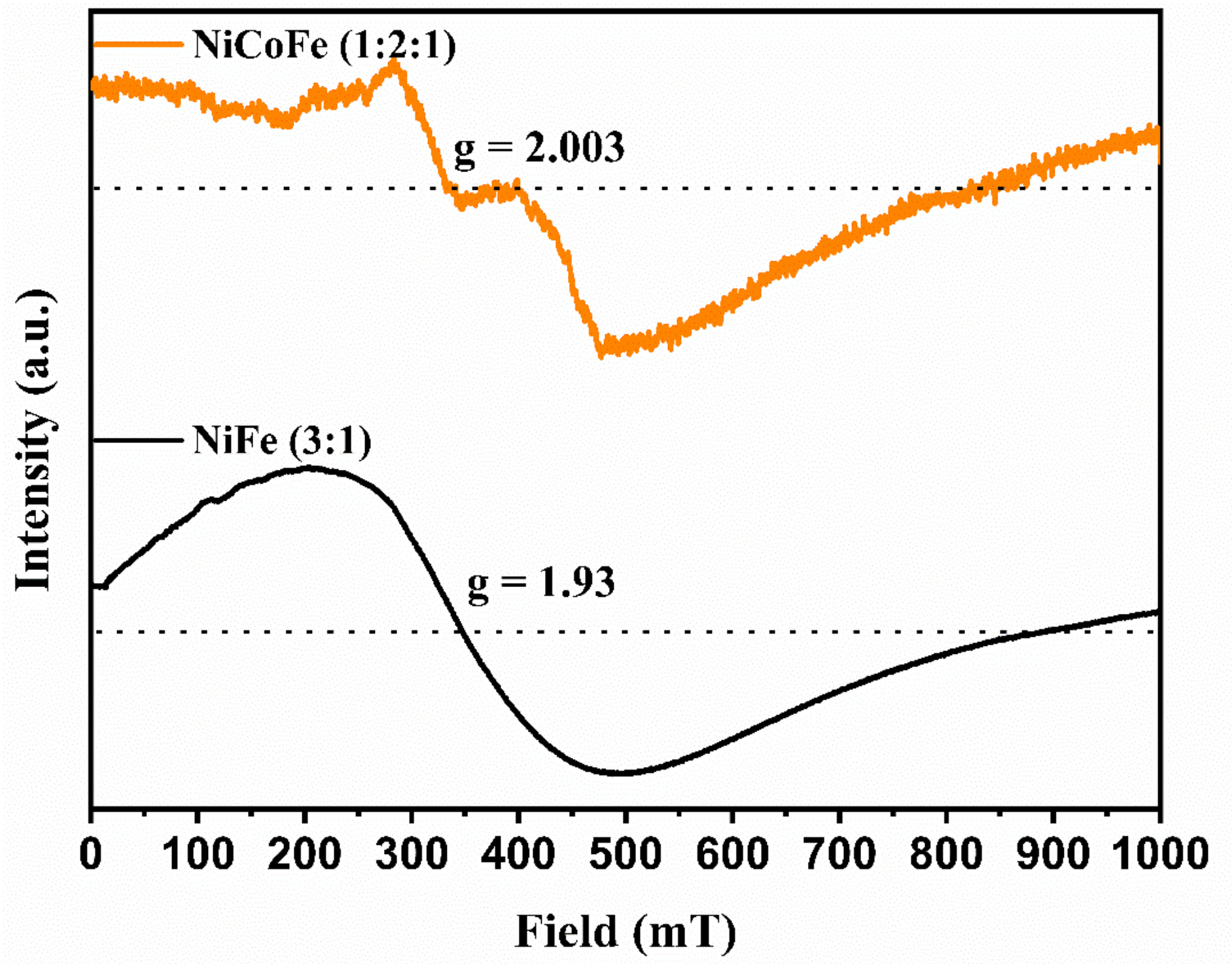

Figure S10. *X-band EPR spectra of pristine NiFe (3:1) and Co-substituted NiCoFe (1:2:1) LDH recorded at a microwave frequency of 9.4 GHz*

Table S5. *ICP–MS-derived elemental weight percentages and experimentally determined metal molar ratios of pristine NiFe (3:1) and Co-modified NiCoFe (1:2:1) LDH compositions.*

Sample	Element	ICP - MS (μg/L)	Elemental wt.%	Metal atomic %	Nominal metal ratio	Experimental metal ratio
NiFe (3:1)	Ni	1297.220	35.97	70.84	3:1	2.43:1
	Fe	507.998	14.09	29.16		
NiCoFe (1:2:1)	Ni	454.613	9.18	15.87	1:2:1	0.57:2.00:1
	Co	1613.416	32.59	56.11		
	Fe	763.434	15.42	28.02		
